# T helper cells with specificity for an antigen in cardiomyocytes promote pressure overload-induced progression from hypertrophy to heart failure

**DOI:** 10.1038/s41598-017-16147-1

**Published:** 2017-11-22

**Authors:** Carina Gröschel, André Sasse, Charlotte Röhrborn, Sebastian Monecke, Michael Didié, Leslie Elsner, Vanessa Kruse, Gertrude Bunt, Andrew H. Lichtman, Karl Toischer, Wolfram-Hubertus Zimmermann, Gerd Hasenfuß, Ralf Dressel

**Affiliations:** 1 Institute of Cellular and Molecular Immunology, University Medical Center Göttingen, Göttingen, Germany; 2 DZHK (German Center for Cardiovascular Research), partner site Göttingen, Göttingen, Germany; 3 Institute of Pharmacology and Toxicology, University Medical Center Göttingen, Göttingen, Germany; 4 Department of Cardiology and Pneumology, University Medical Center Göttingen, Göttingen, Germany; 5 Clinical Optical Microscopy, Department of Neuropathology, University Medical Center Göttingen, Göttingen, Germany; 6 Department of Pathology, Brigham and Women’s Hospital, Harvard Medical School, Boston, MA USA

## Abstract

We investigated whether CD4^+^-T cells with specificity for an antigen in cardiomyocytes promote the progression from hypertrophy to heart failure in mice with increased pressure load due to transverse aortic constriction (TAC). OT-II mice expressing a transgenic T cell receptor (TCR) with specificity for ovalbumin (OVA) on CD4^+^-T cells and cMy-mOVA mice expressing OVA on cardiomyocytes were crossed. The resulting cMy-mOVA-OT-II mice did not display signs of spontaneous autoimmunity despite the fact that their OVA-specific CD4^+^-T cells were not anergic. After TAC, progression to heart failure was significantly accelerated in cMy-mOVA-OT-II compared to cMy-mOVA mice. No OVA-specific antibodies were induced in response to TAC in cMy-mOVA-OT-II mice, yet more CD3^+^ T cells infiltrated their myocardium when compared with TAC-operated cMy-mOVA mice. Systemically, the proportion of activated CD4^+^-T cells with a Th_1_ and Th_17_ cytokine profile was increased in cMy-mOVA-OT-II mice after TAC. Thus, T helper cells with specificity for an antigen in cardiomyocytes can directly promote the progression of heart failure in response to pressure overload independently of autoantibodies.

## Introduction

Heart failure is among the most frequent causes of morbidity and mortality in western countries with an estimated prevalence of more than 37 million individuals globally^1^. It is a highly complex disease, which can result from acute injury, e.g., myocardial infarction or chronic processes such as renal dysfunction, hypertension, or aortic stenosis. Initially, the heart can adapt to volume or pressure overload associated with the chronic diseases, but later the risk of maladaptive remodeling of the myocardium increases and transition from hypertrophy to heart failure occurs. The progression of the disease involves besides myocardial factors such as aberrant calcium handling, apoptosis of cardiomyocytes, and fibrosis also systemic factors including neuro-hormonal activation and inflammation^2^. Inflammation is not restricted to classic inflammatory cardiomyopathies caused by immune responses to infections but also occurs in response to hemodynamic overload^3^. Signs of inflammation have been observed during the progression of chronic heart failure in many clinical studies^4^. In particular, high levels of circulating pro-inflammatory cytokines such as interleukin (IL)-1β, IL-6, and tumor necrosis factor (TNF)-α have been reported in patients and animal models with pressure overload^5,6^. While a deficiency for IL-6 attenuates pressure overload-induced cardiac dysfunction in mice^7^, attempts to employ anti-inflammatory drugs such as infliximab or etanercept, which both target TNF-α, in the therapy of patients with heart failure have been largely unsuccessful^8,9^, possibly due to a functional redundancy of individual cytokines^10^. Therefore, it remains pivotal to gain a better understanding of the role of inflammation^11^ and autoimmunity^3,12,13^ in the pathophysiology of heart failure to identify new therapeutic targets.

Notably, the pathophysiology of volume and pressure overload is remarkably different as murine models of volume (aorto-caval shunt) and pressure overload (transverse aortic constriction, TAC) have shown, in which the same mean total wall stress was achieved by both interventiones^14^. In this study, only TAC was associated with inflammation. At day seven after TAC, a leukocyte infiltration was observed in the myocardium and gene expression data suggested an activation of B and T cell receptor signaling pathways^14^. Only recently, researchers have started to analyze the role of the adaptive immune system in the pathogenesis of pressure overload-induced heart failure in more detail. Laroumanie and colleagues reported that mice deficient for the recombination activation gene 2 (*Rag2*), which do not have B and T cells, were protected from the transition from hypertrophy to heart failure after TAC^15^. This study pointed towards a role specifically for CD4^+^-T helper cells since mice deficient for major histocompatibility complex (MHC) class II molecules, which lack CD4^+^-T helper cells were also protected in contrast to mice devoid of CD8^+^ cytotoxic T cells. Moreover, indirect evidence suggested that the effect of CD4^+^-T helper cells on cardiac function after TAC depends on antigen recognition because T cell receptor (TCR)-transgenic OT-II mice, which possess predominantly CD4^+^-T helper cells specific for ovalbumin (OVA), an antigen absent in mice, did also not show progression into heart failure after TAC^15^. Another study demonstrating that TCRα-deficient mice or mice, in which T cells were depleted by administration of anti-CD3 antibodies, had a preserved cardiac function after TAC^16^, further supports these results. Mice, in which the infiltration of T cells into the myocardium was impaired due to a deficiency of the intercellular adhesion molecule 1 (ICAM1), were also protected from TAC-induced heart failure^17^. Moreover, in line with these data, it has been demonstrated recently that blocking the activation of T cells by abatacept, a cytotoxic T-lymphocyte-associated protein 4 (CTLA4)-Ig fusion protein that blocks T cell co-stimulation, delays progression of TAC-induced heart failure^18,19^.

We have now addressed the question whether CD4^+^-T helper that possess specificity for an antigen expressed in cardiomyocytes are directly involved in the progression from hypertrophy to heart failure due to pressure overload. Since OT-II mice, which have T helper cells with specificity for OVA^20^, were reported to be protected from progression into heart failure after TAC^15^, we asked whether the deterioration of heart function would be accelerated in OT-II mice that express OVA in cardiomyocytes. To obtain such mice, we crossed OT-II with cMy-mOVA mice^21^, which express OVA, fused to the transmembrane part of the human transferrin receptor at the plasma membrane of cardiomyocytes driven by a murine cardiac α-myosin heavy-chain (αMHC) promoter. When the resulting cMy-mOVA-OT-II mice were subjected to TAC, the transition from hypertrophy to heart failure was indeed accelerated but OVA-specific autoantibodies were not induced. Therefore, T helper cells with specificity for an antigen expressed in cardiomyocytes directly exert negative effects on cardiac function in this model.

## Results

### OVA-specific T helper cells are present in high frequency in the spleen of cMy-mOVA-OT-II mice and they can be activated by OVA *in vitro*

**Figure 1:**
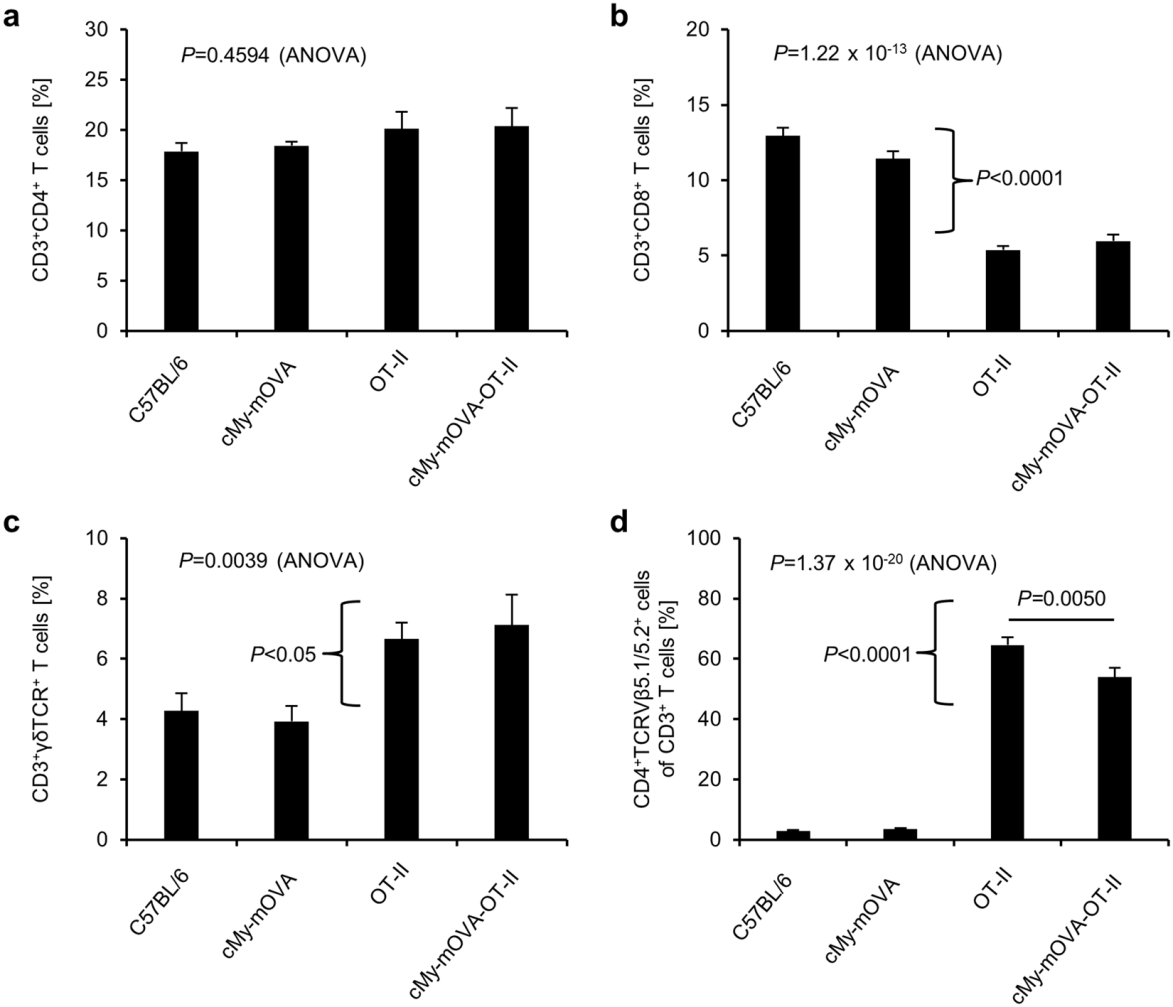
OVA-specific T helper cells are present in high frequency in the spleen of cMy-mOVA-OT-II mice. Splenocytes derived from 8 to 12 weeks old C57BL/6, cMy-mOVA, OT-II, and cMy-mOVA-OT-II mice (n = 8 for each strain) were analyzed by flow cytometry for CD3^+^CD4^+^ T helper cells (**a**), CD3^+^CD8^+^ CTL (**b**), CD3^+^γδTCR^+^ γδT cells (**c**) and the proportion of CD4^+^TCRVβ5.1/5.2^+^ T helper cells among all CD3^+^ T cells (**d**). Means and SEM are shown. The data were analyzed by ANOVA followed by Bonferroni post hoc tests, if significant differences between the mouse strains were revealed. The *P*-values of the ANOVA and of significant post hoc tests are given in the figure. The TCR-transgenic and the TCR-non-transgenic strains differed in addition to the CD4^+^TCRVβ5.1/5.2^+^ T helper cells also in the proportion of CTL and γδT cells present in the spleen and the respective *P*-values of Bonferroni post hoc tests are given beside the brackets.

**Figure 2:**
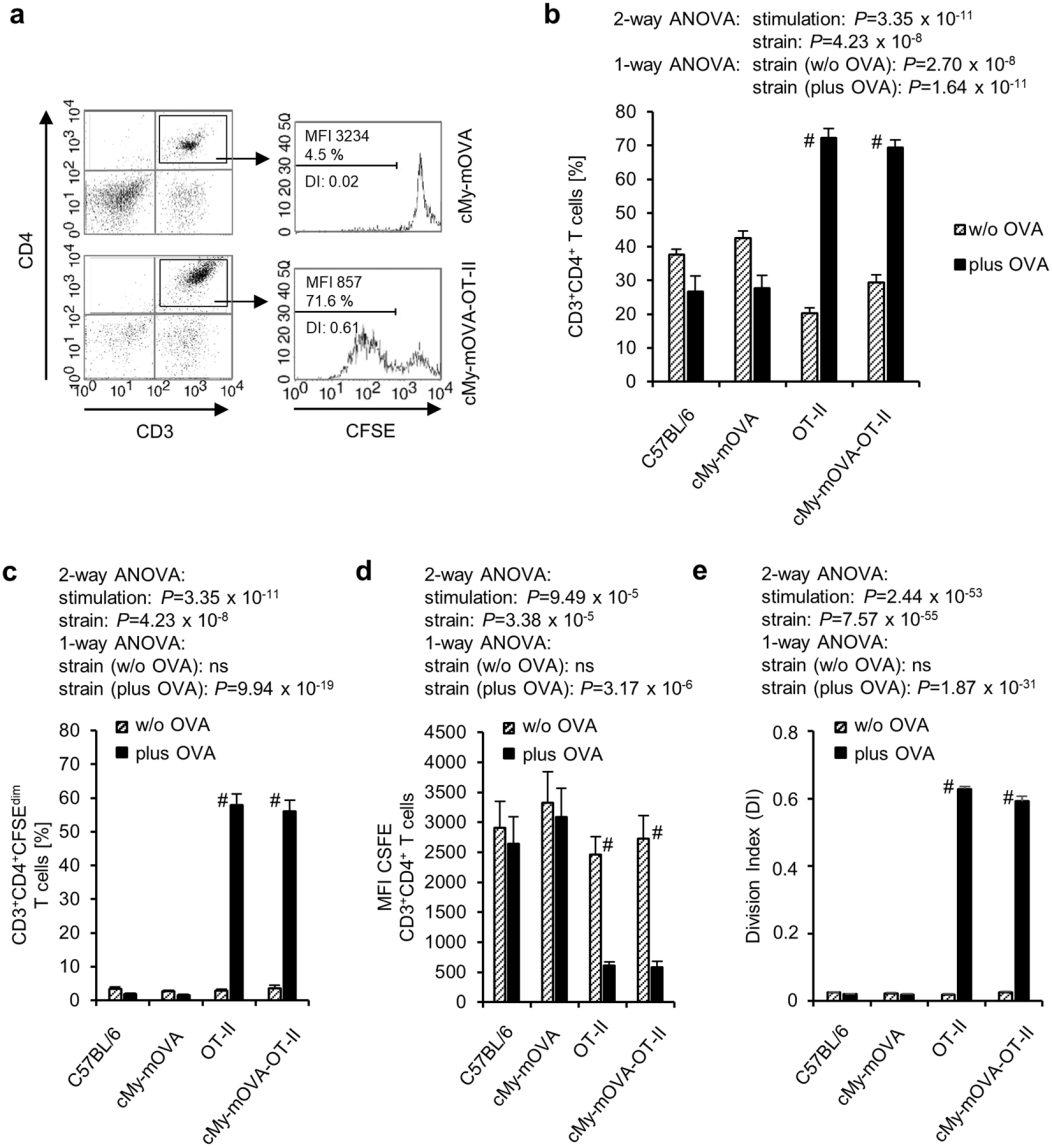
OVA-specific T helper cells of cMy-mOVA-OT-II mice are not anergic and proliferate *in vitro* in response to OVA. Splenocytes derived from 8 to 12 weeks old C57BL/6, cMy-mOVA, OT-II, and cMy-mOVA-OT-II mice (n = 8 for each strain) were stained with CFSE and cultured in absence (w/o OVA) or presence of 250 µM OVA (plus OVA) for 5 days. Afterwards, the cells were stained with anti-CD3 and anti-CD4 antibodies and the CFSE fluorescence of the CD3^+^CD4^+^ T cells was determined by flow cytometry. (**a**) Examples of gating of CD3^+^CD4^+^ T cells and the CFSE fluorescence on CD3^+^CD4^+^ T cells of a cMy-mOVA (upper panels) and a cMy-mOVA-OT-II mouse (lower panels) after culture in presence of OVA are shown. The fractions of proliferating cells with reduced CFSE intensity (CFSE^dim^) were identified in the marked range. (**b**) Means plus standard errors of the mean (SEM) of the proportions of CD3^+^CD4^+^ T cells and (**c**) proliferating T helper cells (CD3^+^CD4^+^CFSE^dim^) are shown. (**d**) The MFI of CFSE of CD3^+^CD4^+^ T cells was determined and means plus SEM are displayed. (**e**) In addition, the division index (DI) of the CD4^+^ T cells has been calculated and is shown as means plus SEM. Effects of the stimulation with antigen (w/o OVA versus plus OVA) and differences between the four mouse strains were analyzed by 2-way-ANOVA and the *P*-values are indicated above the diagrams. In addition, differences between the four mouse strains were analyzed separately for cultures without (strain (w/o OVA)) and with antigen (strain (plus OVA)) by 1-way-ANOVA. If significant differences were revealed, Bonferroni post hoc tests always indicated significant differences (*P* < 0.01) between the TCR-transgenic strains (OT-II and cMy-mOVA-OT-II) and the non-TCR-transgenic strains (C57BL/6 and cMy-mOVA) but none within these groups. Moreover, significant differences between control (w/o OVA) and antigen stimulated (plus OVA) cells within each of the four strains were evaluated by *t*-tests and are indicated in the panels (^#^ *P* < 0.001).

We crossed cMy-mOVA mice with OT-II mice to obtain animals that express ovalbumin (OVA) in cardiomyocytes and have CD4^+^-T cells mostly with specificity for this antigen. These cMy-mOVA-OT-II mice were viable and did not display any overt signs of autoimmunity or heart disease up to an age of more than one year. In accordance to expectations both, cMy-mOVA and cMy-mOVA-OT-II mice expressed OVA in the heart, in contrast to C57BL/6 and OT-II mice (Supplementary Fig. 1). The proportion of T helper cells (Fig. 1a) as well as B cells and NK cells among splenocytes (Supplementary Fig. 2a, b) was similar in C57BL/6, cMy-mOVA, OT-II, and cMy-mOVA-OT-II mice. The percentage of cytotoxic T lymphocytes (CTL) was reduced (Fig. 1b) while γδT cells were slightly more frequent in both T cell receptor (TCR)-transgenic strains (Fig. 1c). On average 54% of the CD3^+^ T cells in the spleens of the cMy-mOVA-OT-II mice were CD4^+^-T helper cells that expressed the transgenic TCR (Vβ5.1/5.2^+^) (Fig. 1d). This proportion was slightly lower than in OT-II mice (65%), but much higher than the percentage of T cells employing the respective variable regions Vβ5.1 or Vβ5.2 of the TCRβ chain in the non-TCR-transgenic strains (3%, C57BL/6 and cMy-mOVA). Thus, OVA-specific T helper cells reached the spleen, a peripheral immunologic organ of the cMy-mOVA-OT-II mice in high numbers but they did not elicit autoimmunity spontaneously. When splenocytes were exposed *ex vivo* to OVA (250 µM for 5 days), CD4^+^-T cells of both OT-II and cMy-mOVA-OT-II mice proliferated vigorously, in contrast to those from non-TCR-transgenic C57BL/6 and cMy-mOVA mice (Fig. 2a–e). This indicates that the OVA-specific T helper cells in the cMy-mOVA-OT-II mice could be activated and were not driven into anergy despite presence of OVA in cardiomyocytes.

### Presence of OVA-specific T helper cells accelerates the progression from hypertrophy to heart failure after TAC in mice that express OVA in cardiomyocytes

**Figure 3:**
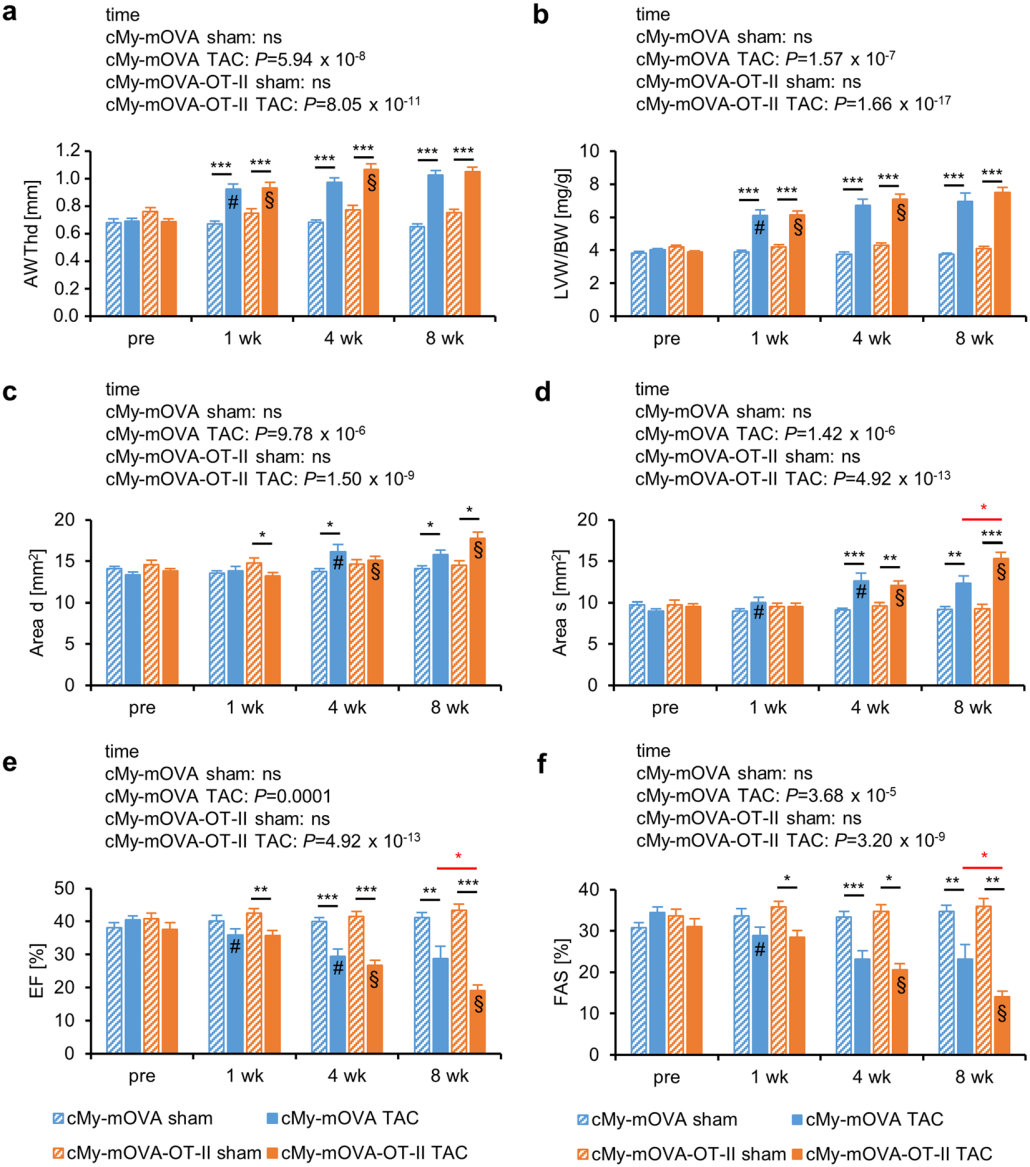
T helper cells accelerate the progression from hypertrophy to heart failure after TAC in cMy-mOVA-OT-II mice. Echocardiography was performed in cMy-mOVA and in cMy-mOVA-OT-II mice before (pre; n = 22 sham and n = 22 TAC for cMy-mOVA and n = 15 sham and n = 22 TAC for cMy-mOVA-OT-II mice) as well as 1 week (n = 22 or 18 for cMy-mOVA and n = 15 or 22 for cMy-mOVA-OT-II), 4 weeks (n = 22 or 17 for cMy-mOVA and n = 15 or 21 for cMy-mOVA-OT-II), and 8 weeks (n = 20 or 11 for cMy-mOVA and n = 15 or 19 for cMy-mOVA-OT-II) after sham or TAC surgery. (**a**) Anterior wall thickness in systole (AWThd), (**b**) left ventricular weight/body weight (LVW/BW) ratio, (**c**) area of the endocardium in diastole (Area d), (**d**) area of the endocardium in systole (Area s), (**e**) ejection fraction (EF), and (**f**) fractional area shortening (FAS) were determined and means plus SEM are displayed. Differences between the time points within each strain were analyzed by a mixed linear model (time) and the *P*-values are given in the panels. Significant differences in the post hoc test (*P* < 0.05) compared to the previous time point are indicated for cMy-mOVA (#) and cMy-mOVA-OT-II mice (§). Differences between sham and TAC groups at a given time point were analyzed by *t*-tests and significant differences are indicated by black bars (*P < 0.05, **P < 0.01,***P < 0.001). Similarly, differences between cMy-mOVA and cMy-mOVA-OT-II mice after TAC were analyzed and red bars and stars indicate significant *P*-values.

Next, we determined whether the presence of T helper cells with specificity for an antigen present in cardiomyocytes promotes the development of heart failure after TAC. Therefore, the cMy-mOVA-OT-II and, as control, cMy-mOVA mice underwent TAC or sham surgery and echocardiography was performed to determine heart function 1, 4, and 8 weeks after the operation. In both strains, the aortic stenosis obtained by TAC was on average similar (Supplementary Fig. 3a). Cardiac hypertrophy developed within 1 week with the anterior wall thickness in diastole (AWTHd) and left ventricular weight/body weight (LVW/BW) ratio increasing as determined by echocardiography (Fig. 3a,b). In cMy-mOVA-OT-II mice, the hypertrophy further progressed from 1 to 4 weeks, whereas it remained stable in cMy-mOVA mice. A dilation of the left ventricle occurred in both strains although the main increase in the area of the endocardium in diastole (Area d) was observed in cMy-mOVA mice from 1 week to 4 weeks and in cMy-mOVA-OT-II mice from 4 weeks to 8 weeks (Fig. 3c). The same pattern was observed when the area of the endocardium in systole (Area s) was analyzed and regarding this parameter, the dilation was at 8 weeks more pronounced in cMy-mOVA-OT-II than cMy-mOVA mice (Fig. 3d). Most importantly, heart function measured as ejection fraction (EF) (Fig. 3e) or fractional area shortening (FAS) (Fig. 3f) exhibited a steeper decline 8 weeks after TAC in cMy-mOVA-OT-II compared to in cMy-mOVA mice. In summary, we observed a faster progression from hypertrophy to heart failure in the cMy-mOVA-OT-II mice that in addition to a model antigen in cardiomyocytes (OVA) possess large numbers of T helper cells with specificity for this antigen. Despite the accelerated progression of heart failure in cMy-mOVA-OT-II mice, their mortality was similar to cMy-mOVA mice (Supplementary Fig. 3b).

### No induction of OVA-specific autoantibodies in cMy-mOVA-OT-II mice

**Figure 4:**
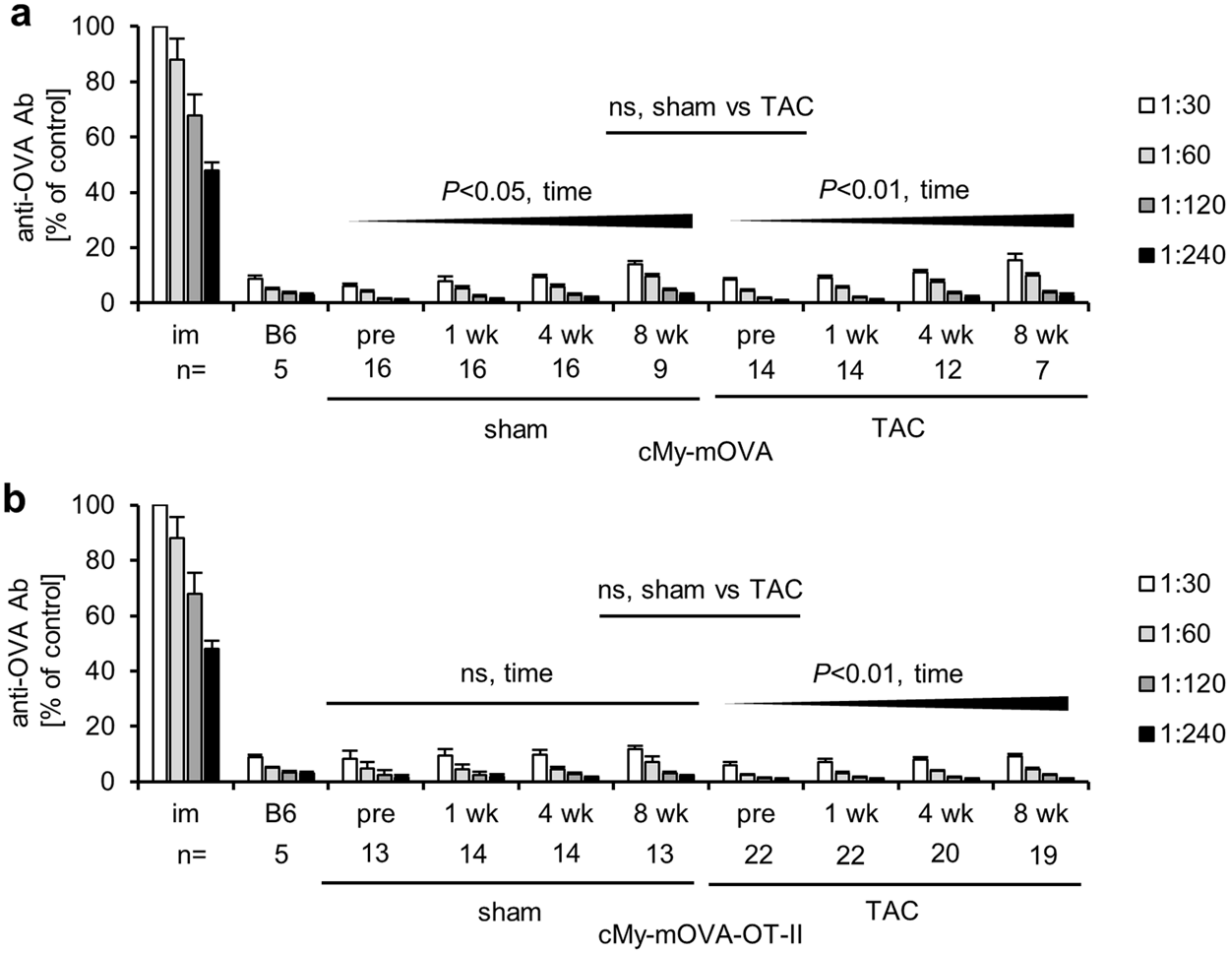
No induction of OVA-specific autoantibodies in cMy-mOVA-OT-II mice after TAC. OVA-specific autoantibodies were determined by ELISA in serial-diluted (1:30, 1:60, 1:120, 1:240) sera of (**a**) cMy-mOVA and (**b**) cMy-mOVA-OT-II mice before (pre) and at 1, 4, and 8 weeks after sham or TAC surgery. Pooled serum (pre-diluted 1:10000) of 4 FVB mice immunized with OVA (im) served as positive control on each plate and the OD at the highest dilution was set to 100% to adjust the other results and means plus SEM are shown. Sera of C57BL/6 (B6) mice (n = 5) served as negative control. The numbers of analyzed animals are indicated. The increase of anti-OVA antibodies over time (pre to 8 weeks) and differences between sham and TAC-operated mice have been analyzed by repeated measures ANOVA and significant *P*-values are indicated (ns, non-significant).

Antibodies against myocardial antigens can contribute to the deterioration of heart function in patients with heart failure^22,23^. Therefore, blood was taken to determine the presence of anti-OVA antibodies before (pre) and 1, 4, and 8 weeks after surgery. Only low amounts of low affinity antibodies were found in sera of cMy-mOVA and cMy-mOVA-OT-II mice similarly to sera of C57BL/6 wild type mice, which served as negative control (Fig. 4a,b). They slightly increased over time (pre-operation to 8 weeks) in TAC and sham-operated cMy-mOVA and TAC-operated cMy-mOVA-OT-II mice. At no time point up to 8 weeks, a significant difference between the sera of TAC and sham-operated cMy-mOVA or cMy-mOVA-OT-II mice was detected. Only in cMy-mOVA mice that were sacrificed at the end of the experiment 10 weeks after sugery, slightly more anti-OVA-antibodies were found in TAC than sham-operated mice (Supplementary Fig. 4a,b). Thus, increasing the cardiac afterload by TAC was not associated with an induction of anti-OVA autoantibodies in cMy-mOVA-OT-II mice (and hardly in cMy-mOVA mice). In summary, it can be concluded that OVA-specific autoantibodies cannot be responsible for the faster progression of heart failure that we observed in cMy-mOVA-OT-II mice.

### Presence of OVA-specific T helper cells leads to increased hypertrophy of cardiomyocytes, decreased capillary density and more perivascular fibrosis 10 weeks after TAC

**Figure 5:**
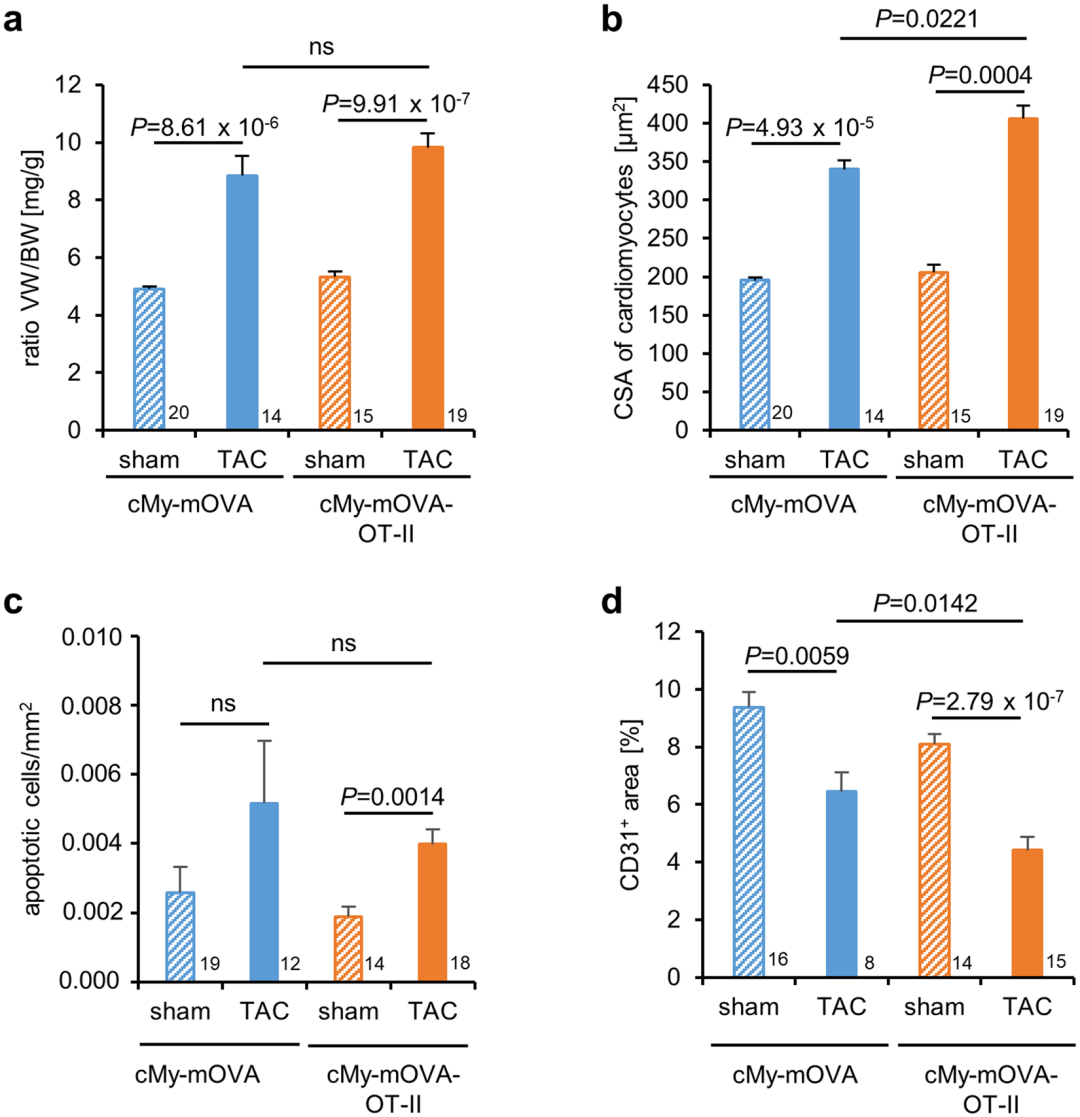
Increased hypertrophy of cardiomyocytes and decreased capillary density in cMy-mOVA-OT-II mice after TAC. The ventricular weight/body weight (VW/BW) ratio (**a**), the CSA of cardiomyocytes (**b**), the number of apoptotic cells positive for cleaved caspase 3 (**c**), and the area of CD31^+^ endothelial cells (**d**), were determined in the myocardium after 10 weeks in sham and TAC-operated cMy-mOVA and cMy-mOVA-OT-II mice and means plus SEM are shown. The numbers of mice analyzed are indicated in the panels beside the bars. Differences between two groups were analyzed by U-tests and significant *P*-values are given in the panels (ns, non-significant).

**Figure 6:**
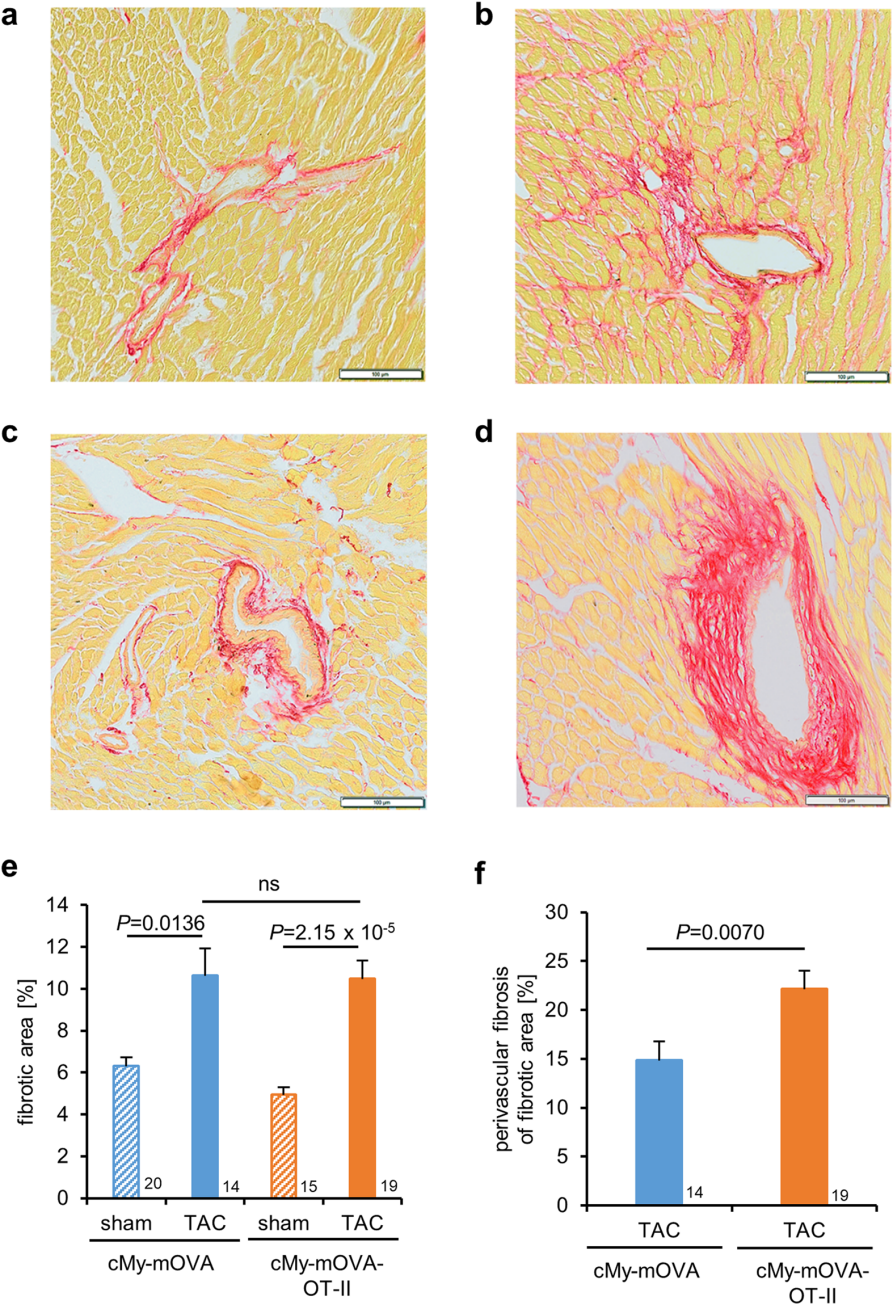
Increased perivascular fibrosis in the myocardium of cMy-mOVA-OT-II mice. Fibrosis of the myocardium was analyzed by Sirius Red staining in cMy-mOVA (**a**,**b**) and cMy-mOVA-OT-II mice (**c**,**d**) after sham (**a**,**c**) and TAC operation (**b**,**d**). The bars indicate 100 µm. The areas containing collagen (**e**) were determined 10 weeks after sham or TAC operation in cMy-mOVA and cMy-mOVA-OT-II mice. In TAC-operated mice, the proportion of perivascular fibrosis (**f**) was analyzed separately. Means plus SEM are shown in panels **e** and **f**. The numbers of mice analyzed are indicated beside the bars. Differences between two groups were analyzed by U-tests and significant *P*-values are given in the panels (ns, non-significant).

The experiment was scheduled to end 10 weeks after the operation (on average on day 69) and hearts and spleens were taken for subsequent analysis. Samples from TAC-operated mice that had to be sacrificed due to clinical symptoms of heart failure but lived longer than 6 weeks were included in these analyzes. At preparation, the ventricular hypertrophy after TAC measured as ventricular weight/body weight (VW/BW) ratio (Fig. 5a) was found to be similar in cMy-mOVA and cMy-mOVA-OT-II mice, which is in accordance with the results of echocardiography (Fig. 3b). However, at the cellular level, the hypertrophy of cardiomyocytes, determined as cross-sectional area (CSA) on slides stained with wheat germ agglutinin (WGA) that marks plasma membranes (Supplementary Fig. 5), was more extensive in cMy-mOVA-OT-II than cMy-mOVA mice after TAC (Fig. 5b). Few apoptotic cells were detected by staining of cleaved caspase 3 in the hearts of sham or TAC-operated mice (Fig. 5c). Hardly any of these were identified as cardiomyocytes (Supplementary Fig. 6), at least at the late time points investigated here (10 weeks after TAC). Overall, more apoptotic cells were present in TAC than sham-operated mice although this difference was statistically significant only in cMy-mOVA-OT-II mice due to considerable individual variations in cMy-mOVA mice. Notably, the capillary density, measured as the proportion of the CD31^+^ endothelial area in the myocardium (Supplementary Fig. 7), decreased in response to TAC in both strains but was significantly more reduced in TAC-operated cMy-mOVA-OT-II than cMy-mOVA mice (Fig. 5d). The degree of fibrosis determined by Sirius Red staining (Fig. 6a–d) was overall increased after TAC to a similar extent in both strains (Fig. 6e). However, the proportion of perivascular fibrosis was higher in TAC-operated cMy-mOVA-OT-II than cMy-mOVA mice (Fig. 6f, Supplementary Fig. 8). These differences in hypertrophy of cardiomyocytes, capillary density and perivascular fibrosis might explain the greater reduction of cardiac function in cMy-mOVA-OT-II compared to cMy-mOVA mice, which was observed in echocardiography.

### Presence of OVA-specific T helper cells leads to more infiltration of CD3^+^ T cells 10 weeks after TAC

**Figure 7:**
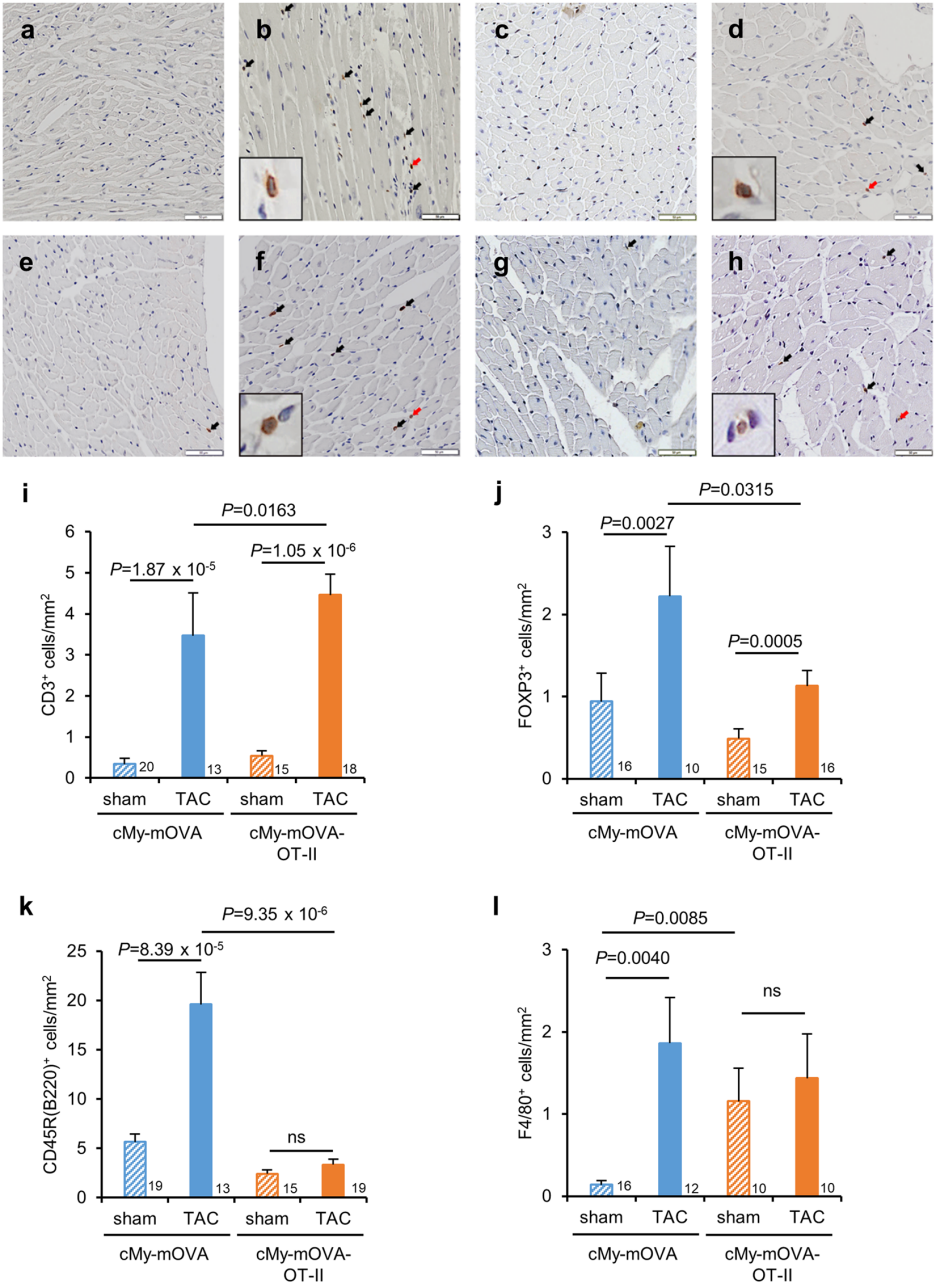
More infiltration of CD3^+^ T cells in cMy-mOVA-OT-II than cMy-mOVA mice after TAC. The infiltration of CD3^+^ T cells (**a**–**d**) and CD45R(B220)^+^ B cells (**e**–**h**) was analyzed by immunohistochemistry in sham (**a**,**e**) and TAC-operated (**b**,**f**) cMy-mOVA mice as well as in sham (**c**,**g**) and TAC-operated (**d**,**h**) cMy-mOVA-OT-II mice. The bars indicate 50 µm. The arrows point to CD3^+^ T cells or CD45R(B220)^+^ B cells, respectively. In panels **b**,** d**, **f**, and **h**, red arrows point towards cells, which are displayed in larger magnification in the inlets of the respective pictures. The numbers of infiltrating CD3^+^ T cells (**i**), FOXP1^+^ regulatory T cells (**j**), CD45R(B220)^+^ B cells (**k**) and F4/80^+^ macrophages (**l**) were determined in the myocardium after 10 weeks in sham and TAC-operated cMy-mOVA and cMy-mOVA-OT-II mice and means plus SEM are shown. The numbers of mice analyzed are indicated in the panels beside the bars. Differences between two groups were analyzed by U-tests and significant *P*-values are given in the panels (ns, non-significant).

Notably, more CD3^+^ T cells were infiltrating the myocardium of cMy-mOVA-OT-II than cMy-mOVA mice after TAC as determined by immunohistochemistry (Fig. 7a–d,i). In contrast, lower numbers of FOXP3^+^ (Fig. 7j) and CD45R(B220)^+^ cells (Fig. 7e–h,k) were present in the myocardium of TAC-operated cMy-mOVA-OT-II than cMy-mOVA mice, suggesting that the infiltration of regulatory T cells (Tregs) and B cells was reduced. Moreover, TAC was also associated with increased numbers of F4/80^+^ macrophages in cMy-mOVA mice. In sham-operated cMy-mOVA-OT-II mice, the number of F4/80^+^ macrophages was higher than in sham-operated cMy-mOVA mice but did not further increase at least at this late time point after TAC (Fig. 7l).

### The proportion of activated T helper cells is increased in cMy-mOVA-OT-II mice after TAC

**Figure 8:**
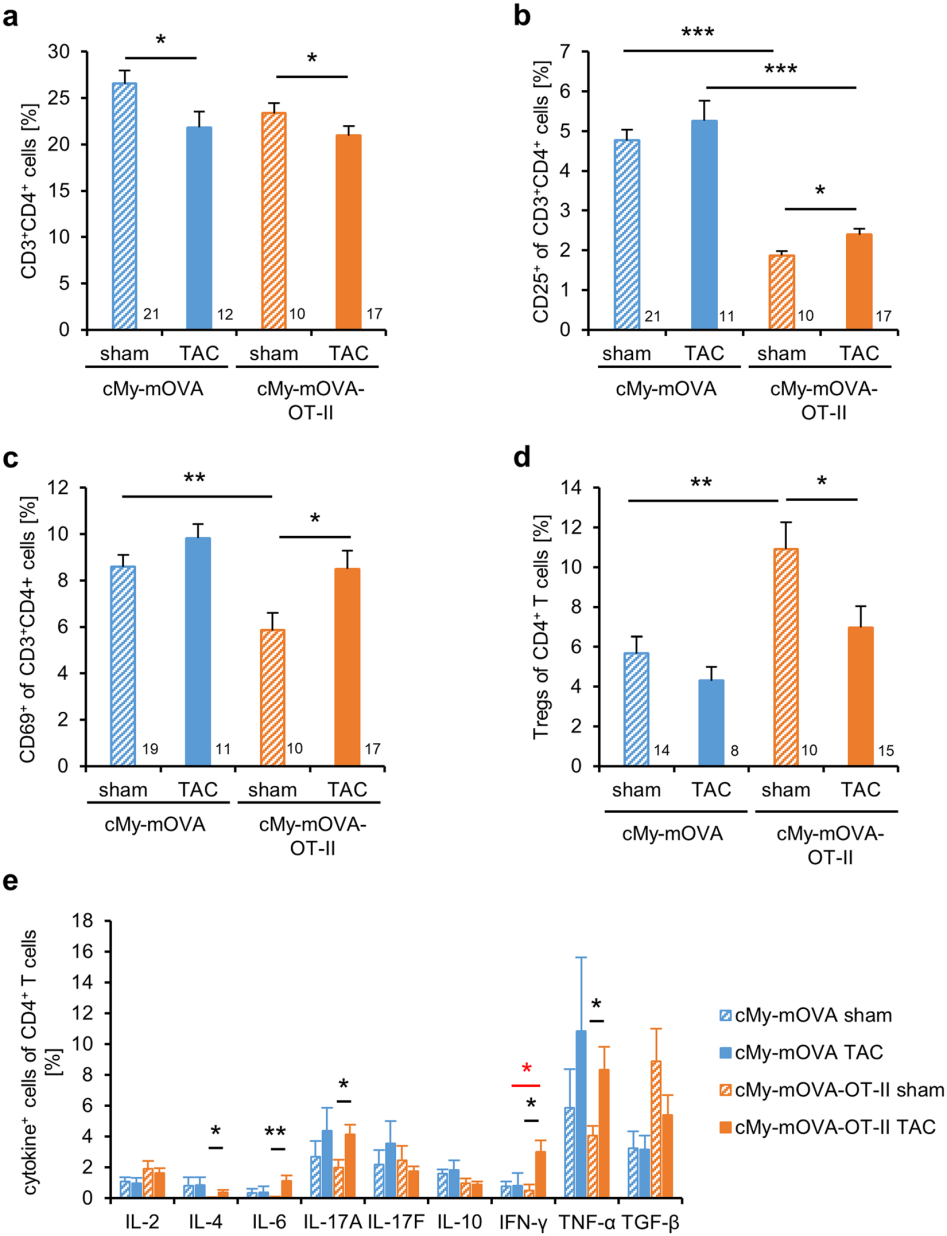
More activated CD4^+^-T cells with a Th_1_ and Th_17_ profile and less regulatory T cells in TAC than sham-operated cMy-mOVA-OT-II mice. (**a**) The proportion of CD3^+^CD4^+^ T helper cells among splenocytes was determined by flow cytometry 10 weeks after sham or TAC operation of cMy-mOVA and cMy-mOVA-OT-II mice. The proportion of activated CD25^+^ cells (**b**) and CD69^+^ cells (**c**) among CD3^+^CD4^+^ T helper cells has been determined in parallel. (**d**) The proportion of CD25^+^FOXP3^+^ regulatory T cells among CD4^+^-T cells was analyzed in addition. (**e**) The proportion of CD4^+^-T cells expressing the indicated cytokines was also determined by flow cytometry directly *ex vivo* without any further stimulation. Means plus SEM are shown and the numbers of mice analyzed are indicated in the panels **a** to **d** beside the bars. In panel **e**, the numbers of analyzed sham and TAC-operated cMy-mOVA and cMy-mOVA-OT-II mice were: 15/8/10/17 for IL-4 and IL-17F; 14/7/10/17 for IL-2, IL-6, IL-10, and IFN-γ; 9/7/10/17 for IL-17A, TNF-α, and TGF-β. Differences between two groups were analyzed by U-tests and significant *P*-values are indicated (*P < 0.05, **P < 0.01, ***P < 0.001).

To determine the systemic activation of T helper cells after TAC, splenic lymphocytes were analyzed by flow cytometry. The proportion of CD3^+^CD4^+^ T helper cells in the spleen was slightly reduced 10 weeks after TAC compared to sham-operated mice in both, cMy-mOVA and cMy-mOVA-OT-II mice (Fig. 8a). Notably, sham-operated cMy-mOVA-OT-II mice had less T helper cells expressing the activation markers CD25 or CD69 than the cMy-mOVA mice (Fig. 8b,c). This might be caused by the condensed T helper cell repertoire of cMy-mOVA-OT-II mice that could hamper their ability to respond to non-OVA antigens. However, after TAC, the proportion of T helper cells expressing these activation markers increased significantly only in cMy-mOVA-OT-II mice (Fig. 8b,c). The proportion of CD25^+^Foxp3^+^ regulatory T cells among the CD4^+^-T cells was significantly higher in sham-operated cMy-mOVA-OT-II than cMy-mOVA mice, but it decreased significantly after TAC only in cMy-mOVA-OT-II mice (Fig. 8d). There was also a trend towards a reduction of transforming growth factor (TGF)-β^+^ cells among the CD4^+^-T cells in TAC-operated cMy-mOVA-OT-II mice (Fig. 8e), which is in accordance with this observation. In cMy-mOVA-OT-II but not in cMy-mOVA mice, the proportion of CD4^+^-T cells with a Th_1_ (interferon (IFN)-γ, TNF-α) and Th_17_ (IL-6, IL-17A) and, at a very low level, Th_2_ profile (IL-4) increased after TAC (Fig. 8e). Notably, more IFN-γ^+^ T helper cells were present in TAC-operated cMy-mOVA-OT-II than cMy-mOVA mice. Thus, the cMy-mOVA-OT-II mice display signs of an activation of T helper cells and a reduction of regulatory T cells after TAC and those T helper cells with a Th_1_ and Th_17_ cytokine profile might directly contribute to the accelerated transition from hypertrophy to heart failure in these mice.

## Discussion

Several recently published studies revealed a critical role of T cells in the progression of pressure overload-induced heart failure^15–18^. However, these studies did not establish whether T cells with specificity for autoantigens in cardiomyocytes become activated in response to an increased cardiac afterload and contribute directly to the deterioration of cardiac function. The assumption that cardio-depressive CD4^+^-T helper cells occur after TAC and act in an autoantigen-specific manner has been previously based solely on indirect evidence^15^. Moreover, T helper cells could respond to antigens primarily present in other cells than cardiomyocytes, e.g., cardiac fibroblasts and the effects could be mediated indirectly by helping autoreactive B cells to differentiate into plasma cells producing antibodies against myocardial antigens^13^. Autoantibodies against β1-adreno-receptors and M2-acetylcholine-receptors were found after TAC in a rat model^24^ and it is meanwhile well established that those autoantibodies can directly impair cardiac function in patients^22,23^. Therefore, these autoantibodies have been accepted as new therapeutic targets^25,26^. Since they possess an IgG isotype, T helper cells must exist, which facilitate immunoglobulin class switching in the autoreactive B cells. In consequence, adverse effects of CD4^+^-T helper cells on cardiac function could be indirect and mediated via such autoantibodies.

To further address these questions, we have generated a new mouse model to investigate the role of T helper cells with specificity for an antigen expressed in cardiomyocytes in the pressure overload-induced transition from hypertrophy to heart failure. For this purpose, two transgenic mouse lines, i.e. OT-II^20^ and cMy-mOVA mice^21^, were crossed to obtain double transgenic cMy-mOVA-OT-II mice, which express OVA at the plasma membrane of cardiomyocytes and have mostly T helper cells with a TCR that has specificity for this antigen. The cMy-mOVA mice have been shown previously to be tolerant to OVA although the transgene is not expressed in the thymus^21^. However, after adoptive transfer of OVA-specific CD8^+^-T cells from OT-I mice in combination with an infection with a vesicular stomatitis virus engineered to express OVA, the mice developed a severe myocarditis^21^.

The double-transgenic cMy-mOVA-OT-II mice developed normally and showed no signs of autoimmunity or other diseases up to an age of more than one year. On average, 54% of the T cells of these mice were CD4^+^-T cells expressing the transgenic TCR. Thus, the OVA-specific T helper cells, which are at risk to become autoreactive in these mice, were not eliminated in the thymus by the mechanisms of central tolerance. This observation can be explained by the lack of expression of the transgene in the thymus that has been demonstrated in the cMy-mOVA mice^21^. In OT-II mice, the proportion of CD4^+^-T cells expressing the transgenic TCR was slightly higher (on average 65% of all T cells) than in cMy-mOVA-OT-II mice. Thus, we cannot exclude the deletion of a minor proportion of TCR-transgenic T helper cells either in the thymus by circulating dendritic cells^27^ or subsequently in the periphery of the cMy-mOVA-OT-II mice. Alternatively, the proportion of TCR-transgenic T cells might be slightly lower due to the hemizygosity of the cMy-mOVA-OT-II mice for the transgenic TCR. However, the still high numbers of OVA-specific T helper cells present in cMy-mOVA-OT-II mice were not driven into anergy due to mechanisms of peripheral tolerance although OVA was expressed in the heart. They could be activated *ex vivo* in co-culture with antigen presenting cells by exposure to OVA similarly as the OVA-specific T helper cells obtained from OT-II mice. Apparently, the OVA-specific T helper cells in cMy-mOVA-OT-II mice do not come into contact with OVA likely, because there is no damage of cardiomyocytes in healthy mice. Thus, they become neither activated nor anergic, turning the cMy-mOVA-OT-II mice in a suitable model to study the response of potentially autoreactive T helper cells in response to myocardial injury such as pressure overload.

When cMy-mOVA and cMy-mOVA-OT-II mice were subjected to TAC, echocardiography showed that a cardiac hypertrophy developed in both strains within one week as it has been observed in C57BL/6 wild type mice in previous experiments^14^. At four weeks, the hypertrophy remained stable in cMy-mOVA mice, whereas it further increased in cMy-mOVA-OT-II mice. The pressure overload resulted eight weeks after TAC in a more pronounced dilation of the left ventricle (Area s) and most importantly in a more severe decline of heart function (EF and FAS) in cMy-mOVA-OT-II than cMy-mOVA mice. Therefore, T helper cells with specificity for an autoantigen expressed in cardiomyocytes appear to promote the decrease of the ejection fraction. However, the cMy-mOVA mice displayed no advantage with respect to survival despite a better-preserved ejection fraction.

At autopsy, 10 weeks after TAC surgery, the cardiac hypertrophy was similar in cMy-mOVA and cMy-mOVA-OT-II mice, which was in accordance with the results of echocardiography since the LVW/BW ratios at 8 weeks were also similar. However, at the level of individual cardiomyocytes, the hypertrophy was more pronounced in cMy-mOVA-OT-II than cMy-mOVA mice since the average CSA of cardiomyocytes was significantly increased. The TAC-operated cMy-mOVA-OT-II mice displayed also more perivascular fibrosis than the cMy-mOVA mice although the fibrotic area was overall similarly enlarged in both strains after TAC. The capillary density decreased in both stains after TAC as previously observed in wild type mice^28^ but it was more reduced in TAC-operated cMy-mOVA-OT-II than cMy-mOVA mice. Increased hypertrophy of cardiomyocytes, augmented perivascular fibrosis, and reduced capillary density could contribute to the more severely reduced cardiac function observed by echocardiography in cMy-mOVA-OT-II mice 8 weeks after TAC. These findings raise the question how CD4^+^-T cells with specificity for an antigen in cardiomyocytes contribute to progression of pressure overload-induced heart failure.

Pressure overload leads to damage of cardiomyocytes as increased numbers of apoptotic cardiomyocytes have been observed previously one week after TAC^14^. Thus, autoantigens, such as OVA in cMy-mOVA-OT-II mice, could be released from dying cardiomyocytes early after TAC. However, at the late time point investigated in this study, i.e. 10 weeks after TAC, apoptosis of cardiac cells was infrequent and apoptosis of cardiomyocytes was rare. An early infiltration of innate immune cells including macrophages and dendritic cells after TAC, has been observed in previous studies^15–18,29,30^. We found increased numbers of F4/80^+^ macrophages even 10 weeks after TAC in cMy-mOVA mice, whereas the number of macrophages in cMy-mOVA-OT-II mice was already higher in sham-operated mice but not further increased after TAC at least at this late time point. A pro-inflammatory cytokine profile and release of danger associated molecular patterns from dying cells has been observed in mouse models and patients with chronic heart failure^5,15,16,18,31^. Therefore, professional antigen presenting cells, which are resident in the myocardium or infiltrate early in response to tissue injury can take up autoantigens and become activated^32^. They are expected to subsequently activate autoantigen-specific T cells, if these autoreactive T cells are present due to an escape of central tolerance in the thymus and if they are in a non-anergic state because of lacking peripheral tolerance for the respective antigens. In agreement with this expectation, we have observed even 10 weeks after the operation and systemically in the spleen higher proportions of CD4^+^-T helper cells expressing activation markers (CD25, CD69) in TAC than sham-operated mice of both strains. In mediastinal lymph nodes of wild type mice, more CD25^+^ or CD44^high^CD69^low^ activated T cells have been found one week or four weeks after TAC in previous studies^17,18^. In cMy-mOVA-OT-II mice, high numbers of non-anergic OVA-specific T helper cells are present of which apparently some become activated and deteriorate heart function. They were present, albeit in low numbers, even systemically, i.e. in the spleen, in contrast to the previous studies that investigated wild type mice and found activated T cells only in heart draining lymph nodes.

Activated autoreactive T helper cells might contribute to the development of heart failure in different ways. Th_2_-polarized T cells could provide help for B cells, which have B cell receptors with specificity for myocardial autoantigens, to differentiate into antibody producing plasma cells and to switch the immunoglobulin class from IgM to IgG. It is well known that autoantibodies of an IgG isotype, e.g., against β1-adreno-receptors and M2-acetylcholine-receptors can occur after TAC^24^ and in patients with heart failure^22,23^. After binding to these receptors, they can directly induce a depression of cardiac function. However, in the cMy-mOVA-OT-II mice no induction of anti-OVA antibodies was observed excluding that OVA-specific T helper cells act indirectly via anti-OVA-antibodies on heart function. Although anti-OVA antibodies, unlike β1-adreno-receptor or anti-M2-acetylcholine-receptor antibodies, would not be expected to interfere adversely with signaling cascades in cardiomyocytes, they could still exert negative effects, e.g., by triggering antibody-dependent cellular cytotoxicity or complement-mediated cytotoxicity.

Alternatively, autoreactive Th_1_ or Th_17_-polarized T cells could directly contribute to the pathogenesis of pressure overload-induced heart failure. In support of this assumption, we found that a higher proportion of systemic CD4^+^-T helper cells had a Th_1_ (IFN-γ, TNF-α) or Th_17_ (IL-17A, IL-6) cytokine profile in TAC-operated cMy-mOVA-OT-II than cMy-mOVA mice. Increased proportions of T helper cells producing the Th_1_ cytokines IFN-γ and TNF-α were found also in the blood of patients with chronic heart failure^33–35^ and increased numbers of IFN-γ-positive CD4^+^-T cells were present in mediastinal lymph nodes after TAC in a previous study^15^. Nevers and colleagues found an increased mRNA expression of the signature transcription factors Tbet and RORγT in left ventricles of wild type mice one week after TAC, which could indicate ongoing Th_1_ and Th_17_ immune responses^18^. The exact role of Th_17_ cells in the pathogenesis of pressure overload remains to be clarified^36^, but Th_17_ cells were reported to be essential in an experimental autoimmune mouse model of dilated cardiomyopathy^37^ and an imbalance of Th_17_ and immunosuppressive regulatory T cells (Tregs) has been observed in mice and patients with chronic heart failure^38,39^. Since TAC was associated with an infiltration of T cells into the myocardium and more T cells infiltrated the myocardium of cMy-mOVA-OT-II than cMy-mOVA mice 10 weeks after TAC, these T cells might directly contribute to the deterioration of heart function. This is in line with previous studies reporting an infiltration of T cells into the myocardium after TAC^15,16,18^ and in patients with NYHA class III to IV non-ischemic heart failure as well as in patients with heart failure due to a dilated cardiomyopathy or aortic stenosis^16,18^. T helper cells infiltrating the myocardium after TAC were shown to have an activated CD44^hi^ phenotype in a previous study^15^. Notably, we found reduced numbers of Tregs, in the myocardium of TAC compared to sham-operated cMy-mOVA-OT-II mice. In view of the essential function of Tregs for peripheral tolerance, this presumably aggravates the risk of cardiac autoimmunity even after the limited tissue injury occurring in the TAC model. The finding that less B cells were infiltrating the myocardium of cMy-mOVA-OT-II than cMy-mOVA mice after TAC was unexpected and is currently not easily explainable. However, it might indicate that less B cells become activated in cMy-mOVA-OT-II mice due to the reduced TCR repertoire of T cells that can provide B cells help and activate them.

The Th_1_ and Th_17_-polarized T helper cells might interact with macrophages and other antigen presenting cells in the myocardium, which have taken-up OVA released from dying cardiomyocytes as these normally do not express MHC class II molecules even under inflammatory conditions^40^. T helper cells might act either directly via secretion of cytokines on cardiomyocytes and other cells of the myocardium such as cardiac fibroblasts or indirectly via an activation of other immune cells such as macrophages. Cytokines can directly impair the function of cardiomyocytes as it has been shown for TNF signaling^41^. Moreover, Th_1_ and Th_17_-polarized T cells have been reported to induce cardiac fibrosis and adverse cardiac remodeling^42^. In a model, in which Th_1_-polarized T cells have been induced by TAC, T cells were reported to promote collagen fiber formation by stimulating lysyl oxidase (LOX), an enzyme responsible for collagen cross-linking^15,33^. Nevers and colleagues recently reported that Th_1_ cells, which become activated due to pressure overload, promote cardiac fibrosis in an IFN-γ-dependent manner by inducing the transition of cardiac fibroblasts into TGF-β-producing myofibroblasts^43^. Th_17_-polarized CD4^+^-T cells, on the other hand, may contribute to cardiac fibrosis by secretion of IL-13 and IL-17. Tregs could decrease the risk of fibrosis by anti-inflammatory effects although they produce themselves TGF-β, a potent inducer of fibrosis^42^. Notably, the adoptive transfer of Tregs has been reported to attenuate angiotensin II and TAC-induced cardiac fibrosis^44,45^ and a Treg deficiency in patients has been associated with chronic heart failure^46^. In our experiments, the extent of fibrosis was overall not different 10 weeks after TAC in cMy-mOVA and cMy-mOVA-OT-II mice but the proportion of perivascular fibrosis was increased in the cMy-mOVA-OT-II mice. This is in line with results of other studies demonstrating effects of CD4^+^-T cells mainly on perivascular fibrosis after TAC^43^. Moreover, we cannot exclude that an earlier onset of fibrosis might have contributed to the earlier progression into heart failure in cMy-mOVA-OT-II mice. In T and B cell deficient as well as in CD4^+^ but not in CD8^+^-T cell-deficient mice, fibrosis was significantly reduced after TAC^15^. Similar observations have been made in TCRα-deficient mice and in mice depleted of T cells by administration of anti-CD3 antibodies^16^ as well as in mice in which the T cell infiltration was diminished due an ICAM1-deficiency^17^. Blocking T cell activation by co-stimulation blockade with abatacept 2 days or even 2 weeks after TAC was also reported to reduce cardiac fibrosis^18^. Although the data collectively argue for detrimental effects of T helper cells in pressure overload, it should be noted that T cells might have also beneficial effects in response to cardiac injury. After myocardial infarction, e.g., CD4^+^-T cells were reported to stimulate collagen matrix formation and thereby improve wound healing and survival by reducing the risk of myocardial rupture^47^.

In conclusion, we have shown that autoreactive T helper cells with specificity for an antigen expressed in cardiomyocytes can promote the progression from hypertrophy to heart failure in response to pressure overload. In the cMy-mOVA-OT-II mice, they do this independently of autoantibodies, although the presence of cardiac autoantibodies of the IgG isotype in other animal models and in patients demonstrates that autoreactive T helper cells can act in this manner. In cMy-mOVA-OT-II mice, autoreactive Th_1_ and/or Th_17_-polarized cells act presumably via cytokines directly on cells of the myocardium. In accordance with this data, it recently has been shown in the TAC model that deletion of T cells or blocking T cell activation are new options to treat pressure overload-induced heart failure^16,18^. It remains to be shown, which physiological autoantigens are recognized by T helper cells in these animal models or in patients. In experimental autoimmune myocarditis, e.g., myosin and troponin I can act as autoantigens for T cells since the adoptive transfer of T cells from mice with active myocarditis was sufficient to elicit the disease in the recipients^48,49^. Interestingly, the central tolerance to α-myosin is impaired in mice and man^50^ so this is an obvious candidate antigen. However, in humans, no relevant T cell autoantigens have been definitively identified so far^51^. Very recently, it has been shown that CD4^+^-T cells contribute to age-related myocardial inflammation and functional decline in aged mice^52^. Thus, it should be further explored whether a CD4^+^-T cell-mediated autoimmunity contributes to the progression of heart failure in patients or a subgroup of patients suffering from pressure overload due to age-related diseases that are normally not considered to be associated with autoimmunity such as aortic stenosis and hypertension.

## Materials and Methods

### Mice

C57BL/6, TCR-transgenic OT-II^20^, OVA-transgenic cMy-mOVA^21^, and double transgenic cMy-mOVA-OT-II mice were bred in the central animal facility at the University Medical Center Göttingen under specific pathogen-free conditions in individually ventilated cages and in a 12 h light-dark cycle. Both parental transgenic strains, OT-II and cMy-mOVA, have a C57BL/6 background. Notably, C57BL/6 mice are resistant to experimental autoimmune myocarditis induced by α-myosin as well as to post-infection chronic myocarditis^3^. OT-II and cMy-mOVA mice being homozygous for the transgenes were crossed to obtain cMy-mOVA-OT-II mice that were hemizygous for both transgenes. All animal experiments were approved by the responsible agency (Niedersächsisches Landesamt für Lebensmittelsicherheit und Verbraucherschutz) and were carried out in compliance with German and European legislation (Directive 2010/63/EU).

### Design of animal experiments

Mice of an age between 8 and 12 weeks were used for experiments. Male and female mice were equally distributed among the experimental groups. Beyond that, the mice were randomly assigned to the experimental groups. Our hypothesis being that the heart function could be more impaired in TAC-operated cMy-mOVA-OT-II than cMy-mOVA mice, we wanted to be able to detect at least a reduction of the ejection fraction 8 weeks after TAC from an estimated average of 30% in cMy-mOVA to 20% in cMy-mOVA-OT-II mice with an estimated standard deviation of 8%-points. The required sample size of 11 mice for an analysis in a two-sided *t*-test was calculated by setting the type I error 0.05 and the desired power to 0.80. Moreover, we estimated that 40% (i.e. 7) of the TAC-operated mice could drop out of the experiments earlier than 8 weeks after surgery^14^ and that, in addition, 20% (i.e. 4) of mice could die during surgery or within the first 3 days after TAC or sham operation due to perioperative complications. Therefore, we estimated the minimal number of required mice as 22 for TAC and 15 for sham-operated mice. However, also 22 sham-operated cMy-mOVA mice were available for analysis. Only mice surviving the first 3 days were included in the survival analysis because an earlier lethality is most likely unrelated to heart failure. All animals alive were subjected to echocardiography 1, 2, and 8 weeks after surgery. At these time points, blood was taken to determine anti-OVA antibodies, but from some mice at some time points, not enough blood was obtained to perform the analysis. The post-mortem analyses were performed on heart and spleen samples of mice sacrificed at the planned end of the experiments 10 weeks after surgery and on 3 animals that had to be scarified earlier due to their clinical symptoms. We did not include samples from mice that survived less than 6 weeks to avoid confounding the results with data from animals in an earlier stage of the disease. We could not always perform every measurement on all samples of each animal for various reasons but no data obtained were removed from the analysis.

### Surgery and autopsy

TAC was performed using a minimally invasive operation as described previously^14,53^. Briefly, 8 to 12 weeks old mice were anesthetized by intraperitoneal injections of medetomidin (0.5 mg/kg), midazolam (5 mg/kg), and fentanyl (0.05 mg/kg). After horizontal incision at the jugulum, the transversal aorta was displayed and a 26-gauge needle was tied against the aorta. In sham-operated mice, the surgical thread was not tied. After removal of the needle, the skin was closed and the anesthesia was reversed by subcutaneous injection of atipamezol (2.5 mg/kg) and flumazenil (0.5 mg/kg). For further analgesia, the mice received buprenorphine (60 µg/kg) subcutaneously one hour after surgery. Metamizole (1.33 mg/ml) had to be added to the drinking water for one week to achieve long-term analgesia. Three days after surgery, the pressure gradient over the aortic ligature was determined using pulsed wave Doppler. For echocardiography at 1, 4, and 8 weeks after the operation, the mice were anesthetized with 3-% isoflurane, and temperature, respiration, and ECG-controlled anesthesia was maintained with 1.5-% isoflurane. After each echocardiography, approximately 50 µl blood were taken from the orbital sinus of the mice to measure anti-OVA antibodies in the sera. At the end of the experiments, 10 weeks after the operation, the mice were sacrificed in isoflurane anesthesia (5-%) by cervical dislocation. Blood was taken before the hearts were excised, perfused with saline solution and after weighting of the ventricles, they were immediately processed for histology and immunohistochemistry. Finally, the spleens were harvested and stored in Dulbecco’s modified Eagle medium (DMEM) on ice.

### Echocardiography

Transthoracic echocardiography was performed blinded to the treatment of the mice using a Vevo2100 (VisualSonics, Toronto, Canada) system with a 30 MHz center frequency transducer. B-mode recordings^14,53^ were used to determine the long axis in systole (Ls) and diastole (Ld), the end-diastolic (LVIDd) and end-systolic (LVIDs) left ventricular (LV) chamber diameter and the anterior and posterior wall thickness in systole (AWThs and PWThs) and diastole (AWThd and PWThd), the area of the endocardium in systole (Area s) and diastole (Area d) and the area of the epicardium in systole (Epi s). The evaluation was done blinded to the strain and treatment of the mice. Fractional area shortening (FAS) was calculated as (Area d − Area s)/Area d × 100. The ejection fraction (EF) was calculated as ((5/6) × Area s × Ls) − ((5/6) × Area d × Ld)/((5/6) × Area d × Ld) × 100. Echocardiographic LV weight (LVW) was estimated using the formula: 1.05 × (5/6) × (Epi s × (Ls + (AWThs + PWThs)/2) − (Area d × Ls)).

### Enzyme-linked immuno-sorbent assay (ELISA)

To measure anti-OVA antibodies, 96-well Nunc MaxiSorp microtiter plates were coated overnight at 4 °C with 20 µg/ml OVA (Sigma, Traufkirchen, Germany) in sodium carbonate buffer (pH 8.5; 50 µl/well). After blocking with 1% gelatin in phosphate-buffered saline (PBS), 1:30, 1:60, 1:120, and 1:240 diluted sera (50 µl/well) were added and the plates were incubated for 1 h at 37 °C. On each plate, 1:10000 pre-diluted pooled serum from four FVB/N mice that had been immunized with OVA was added in duplicates as positive control and wells without serum served as blank controls. Moreover, sera from five C57BL/6 were used as negative control. For detection, goat anti-mouse horseradish peroxidase (HRP)-conjugated polyclonal antibodies (115-035-003, Jackson laboratories, via Dianova, Hamburg, Germany) diluted 1:4000 in PBS with 0.05% Tween-20 were used. These antibodies react to mouse immunoglobulins of both IgG and IgM isotypes. After incubation (1 h at 37 °C), 50 µl 2,2′-azino-bis(3-ethylbenzothiazoline-6-sulphonic acid) (ABTS) substrate solution were added to each well and the optical density was determined after 5 min using a BioTek PowerWave 340 microplate spectrophotometer (BioTek, Bad Friedrichshall, Germany) set to 405 nm. The OD of the positive control at the lowest dilution of the positive control was set to 100% and the other results were adjusted accordingly to allow for the comparison of sera analyzed on different plates.

### Histology and immunohistochemistry

Hearts were perfused *ex vivo* via the aorta with 0.9% NaCl before one-third of the heart from the middle part was fixed in 3.7% formaldehyde solution overnight and the other two thirds oriented towards the basis and the apex of the heart were snap frozen in liquid nitrogen. The formaldehyde-fixed samples were embedded in paraffin before 5 µm sections were cut that were used for most analyses. Frozen sections of 7 µm were obtained from samples oriented towards the basis of the hearts to determine the infiltration of FOXP3^+^ cells and CD31^+^ endothelial areas. Collagen was visualized by Sirius Red staining as described previously^53^ to determine the extent of fibrosis. In addition to the total amount of the fibrotic area in the left ventricle, perivascular fibrosis was determined defined as fibrotic area directly associated with vessels of a diameter greater than 100 µm^54^. Infiltration of the myocardium by immune cells was determined by immunohistochemistry as described elsewhere^40^ using anti-CD3 (1:200, MCA1477, rat IgG_1_, ABD Serotec, Oxford, UK), anti-CD45R/B220 (1:200, RA3-6B2, rat IgG_2a_, Biolegend, Fell, Germany), anti-FOXP3 (1:100, with 0.5% Triton-X100, FJK-16s, rat IgG_2a_, eBiosciences, Frankfurt, Germany), and anti-F4/80 monoclonal antibodies (1:200, A3-1, rat IgG_2b_, Biolegend), respectively. For CD3 and CD45R/B220 staining, antigen retrieval was performed by boiling the slides 5 times for 5 min in sodium citrate buffer (10 mmol/L sodium citrate, pH 6, 0.05% Tween 20). Polyclonal biotinylated goat anti-rat IgG secondary antibodies (1:200, 112-065-062, Jackson laboratories) and HRP-conjugated streptavidin (405210, Biolegend) served as secondary and tertiary reagents for the anti-CD3, anti-CD45R/B220, and anti-F4/80 antibodies. The anti-FOXP3 antibody was detected on frozen sections by HRP-conjugated goat anti-rat IgG antibodies (1:200, 112-035-167, Dianova). Endothelial cells were also detected on frozen sections using an antibody against CD31 (1:50, PECAM-1, SC-376764, mouse IgG_1_, Santa Cruz Biotechnology, Heidelberg, Germany) followed by HRP-conjugated goat anti-mouse IgG antibodies (1:200, 115-035-003, Dianova). Apoptotic cells were detected by immunohistochemistry for cleaved caspase 3 using the SignalStain apoptosis detection kit (#12692, Cell Signaling Technology, Leiden, The Netherlands) according to the manufacturer′s instructions. The slides were scanned with a 20x objective (UPlanApo, NA 0.75) using the dotSlide SL slide scanner (Olympus, Hamburg, Germany) equipped with a peltier-cooled XC10 camera. A scientist blinded to the strain and treatment of the mice quantified the extent of fibrosis and the numbers or areas of stained cells in two complete heart sections using cellSens Dimensions software (Olympus). Fibrotic areas and the areas of CD31^+^ endothelial cells were always determined together with the areas of cardiomyocytes. These areas are specified as the proportion of the area of interest relative to the sum of the area of interest and the area of cardiomyocytes. To determine the CSA of individual cardiomyocytes, slides were fluorescently stained for 1 h at room temperature with Alexa680-labeled WGA (5 µg/ml in PBS) (W32465, Invitrogen, Karlsruhe, Germany) to detect plasma membranes, washed two times in PBS and once in water before being mounted with anti-fading mounting medium containing 4′,6-diamidino-2-phenylindole (DAPI) (SCR-038448, Dianova). The slides were then scanned with a 20x objective ((UPlanSApo, NA 0.75) using the virtual slide microscope VS120-L100 (Olympus) equipped with a peltier-cooled XM10 monochrome Camera and the X-Cite excitation source. Alexa680 fluorescence was detected using the excitation filter 650/13 nm and emission filter 684/24 nm with corresponding dichroic mirror. The CSA of at least 10 randomly selected cardiomyocytes was determined in at least two areas from the left ventricle containing cross-sections using the cellSens Dimensions software and the results were averaged per animal.

### T cell stimulation

Lymphocytes were obtained from spleens of C57BL/6, OT-II, cMy-mOVA, and cMy-mOVA-OT-II mice using a Tenbroeck homogenizer. Erythrocytes were removed from splenocytes by incubation for 5 min in lysis buffer (155 mM NH_4_Cl, 10 mM KHCO_3_, 0.1 mM EDTA, pH 7.4–7.8). To allow for the flow cytometric measurement of antigen-induced proliferation, the cells were incubated for 5 min with 5 µM of the dye carboxyfluorescein succinimidyl ester (CFSE) (C-1157, Invitrogen) in phosphate buffered saline PBS/0.1% bovine serum albumin at 37 °C. After being washed 3 times with DMEM containing 10% fetal calf serum (FCS), the splenocytes were cultured in round-bottomed microtiter plates in DMEM/10% FCS with or without 250 µM OVA. After 5 days, the cells were harvested and stained by anti-CD3 and anti-CD4 antibodies, before the CFSE dilution, i.e. the proliferation of T helper cells, was analyzed by flow cytometry. The division index (DI), which is the average number of cell divisions that a cell in the original population has undergone, has been calculated using the FlowJo software (Ashland, OR, USA).

### Flow cytometry

Flow cytometry was performed as described previously^55^ on a FACS Calibur flow cytometer (BD Biosciences, Heidelberg, Germany) using CellQuestPro data acquisition and analysis software. The antibodies used for flow cytometry and isotype controls are listed in Supplementary Table 1. For staining of cell surface antigens, 5 × 10^5^ cells were incubated in 100 μl PBS with 1 μg of the respective primary monoclonal antibody for 30 min at 4 °C before washing with PBS. To stain for intracellular cytokines, splenocytes were stained at 4 °C with anti-CD3 and anti-CD4 antibodies before fixation and permeabilization with Cytofix/Cytoperm and Perm/Wash solutions (BD Biosciences) according to the manufacturer᾽s protocol. For the detection of FOXP3^+^ regulatory T cells, a FOXP3 staining kit (320019, Biolegend) was used after staining of CD4 and CD25 at the plasma membrane. TGF-β was detected on the plasma membrane of CD3^+^CD4^+^ T cells.

### Quantitative polymerase chain reaction (qPCR)

RNA was obtained from hearts of C57BL/6, OT-II, cMy-mOVA, and cMy-mOVA-OT-II mice and transcribed into cDNA, which was used to analyze the expression of *Ova* mRNA by qPCR as described previously^56^ using primers for *Ova* (forward: 5′-AGT GGC ATC AAT GGC TTC T-3′, reverse: 5′-GTT GAT TAT ACT CTC AAG CTG CTC A-3′)^21^ and the housekeeping gene *Gapdh* (forward: 3′-TGT GTC CGT CGT GGA TCT GA-3′, reverse: 5′-TTG CTG TTG AAG TCG CAG GAG-3′). The Δct values (ct *Ova* minus ct *Gapdh*) were used to calculate the relative expression of *Ova* in single heart samples in reference to the mean of the Δct values for the hearts of cMy-mOVA mice (Δct reference) (2^Δct *Ova*^/2^Δct reference^)^57^.

### Statistics

Results are shown as mean with standard error of the mean (SEM). The data were evaluated with the SPSS software (IBM, Armonk, NY, USA). Analyses of variance (ANOVA) was used to compare data sets with more than one experimental group. Repeated measures ANOVA and mixed linear models with the specification auto-regressive process AR (1) were employed to analyze alterations over time, e.g. in echocardiography data sets. Data of two groups were compared by *t*-test or the non-parametric Mann-Whitney U-test, if the values were not normally distributed according to the Kolmogorov-Smirnov test. If the Levene test indicated inequality of variances, an unequal variance *t*-test has been used instead of Student’s *t*-test. The survival curves of TAC-operated mice were compared by a Log Rank (Mantel-Cox) test. *P*-values of <0.05 in two-sided tests was considered to be significant.

### Data availability

The data that support the findings of this study are available from the corresponding author upon reasonable request.

## Electronic supplementary material

Supplementary Materials
