# Supplementary material for: T helper cells with specificity for an antigen in cardiomyocytes promote pressure overload-induced progression from hypertrophy to heart failure
Source: Sci Rep. 2017 Nov 22;7:15998. doi: 10.1038/s41598-017-16147-1 (PMC5700082; doi:10.1038/s41598-017-16147-1)
Supplement: Supplementary file 1 — Supplementary Materials [file 41598_2017_16147_MOESM1_ESM.pdf]

Supplementary Materials

T helper cells with specificity for an antigen in cardiomyocytes promote pressure overload-induced progression from hypertrophy to heart failure

Carina Gröschel, André Sasse, Charlotte Röhrborn, Sebastian Monecke, Michael Didié, Leslie Elsner, Vanessa Kruse, Gertrude Bunt, Andrew H. Lichtman, Karl Toischer, Wolfram-Hubertus Zimmermann, Gerd Hasenfuß, Ralf Dressel

Supplementary Figures 1 to 8

Supplementary Table 1

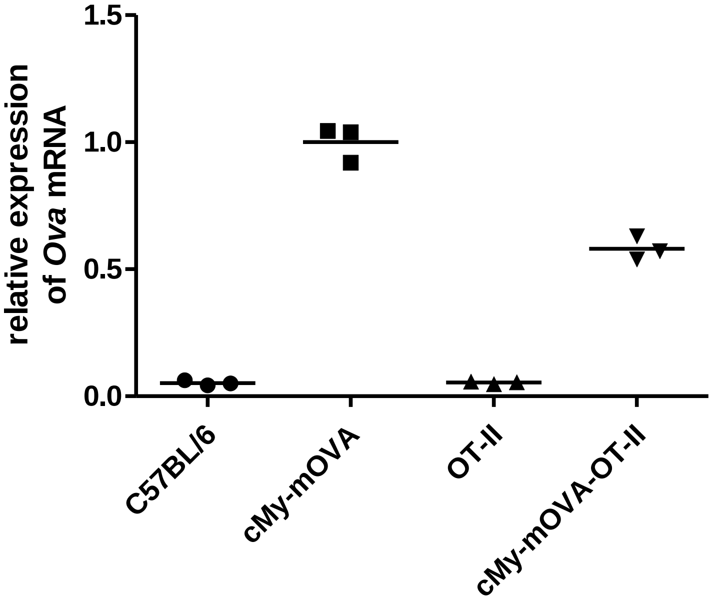

Supplementary Figure 1. *Ova* mRNA is expressed in the hearts of cMy-mOVA and cMy-mOVA-OT-II but not in C57BL/6 and OT-II mice. The mRNA expression was determined in hearts of mice of these strains (n=3) by qPCR in comparison to the house keeping gene *Gapdh*. The Δct values (ct *Ova* minus ct *Gapdh*) were used to calculate the relative expression of *Ova* in single heart samples in reference to the mean of the Δct values for the hearts of cMy-mOVA mice (Δct reference) ($2^{\Delta ct\ Ova}/2^{\Delta ct\ reference}$). Single data points and their means are shown. An ANOVA indicated significant differences between the strains (P=8.03 x 10^{-9}) and Bonferroni post hoc tests revealed differences (P<0.001) between all strains with exception of the comparison of C57BL/6 and OT-II mice. The cMy-mOVA mice were homozygous for the *Ova* transgene whereas cMy-mOVA-OT-II were hemizygous explaining the difference in *Ova* expression levels between these strains.

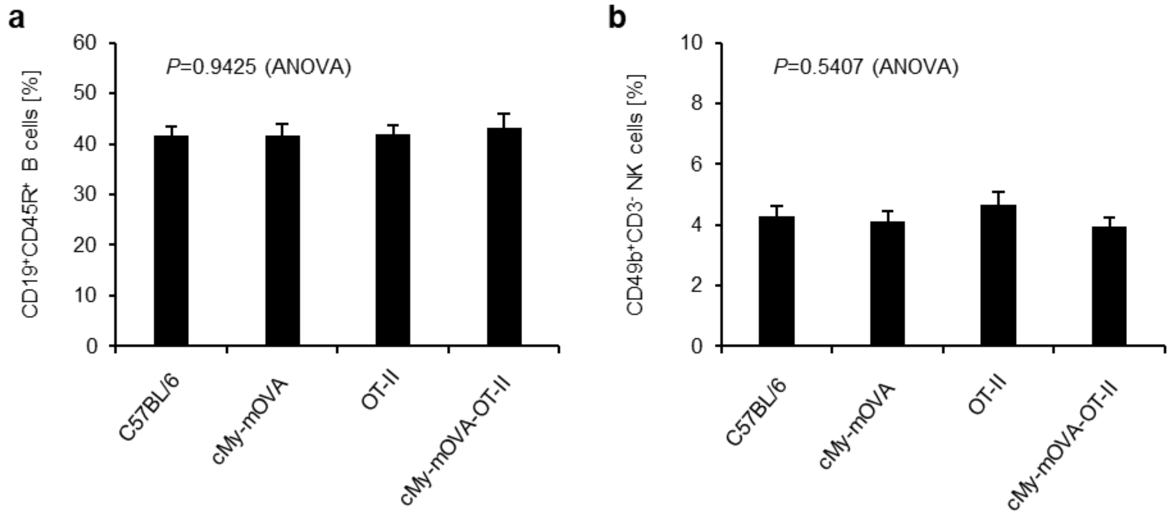

Supplementary Figure 2. The frequency of B cells and NK cells is not altered in the spleen of cMy-mOVA-OT-II mice. Splenocytes derived from 8 to 12 weeks old C57BL/6, cMy-mOVA, OT-II, and cMy-mOVA-OT-II mice (n=8 for each strain) were analyzed by flow cytometry for $CD19^+CD45R^+$ B cells (**a**) and $CD49b^+CD3^-$ NK cells (**b**), Means and SEM are shown. The data were analyzed by ANOVA and the *P*-values are given in the figure.

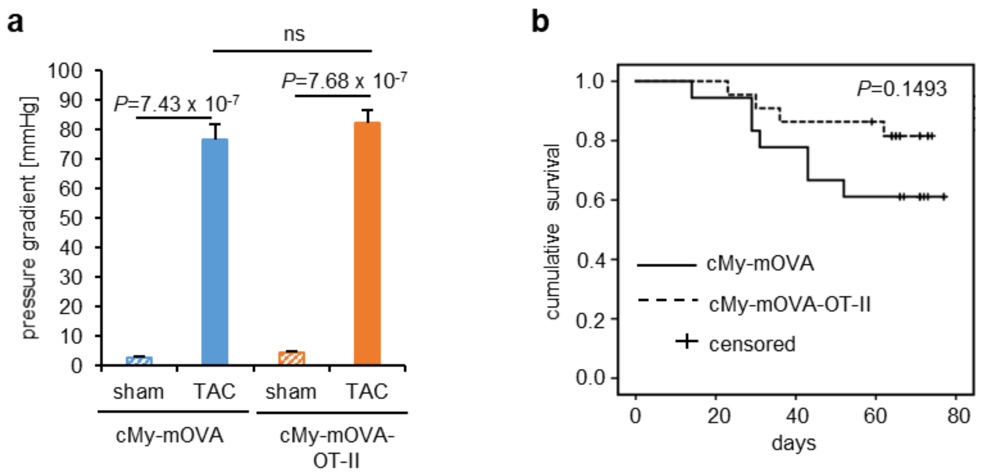

Supplementary Figure 3. The aortic stenosis after TAC operation was similar in cMy-mOVA and cMy-mOVA-OT-II mice and the survival of the animals was not different. (**a**) The pressure gradient over the aortic ligature was determined 3 days after surgery using pulsed wave Doppler in sham (n=22) and TAC-operated (n=18) cMy-mOVA mice as well as in sham (n=15) and TAC-operated (n=22) cMy-mOVA-OT-II mice. Means plus SEM are shown. Differences between two groups were analyzed by U-tests and significant *P*-values are indicated (ns, non-significant). (**b**) Kaplan-Meier survival curves for TAC-operated cMy-mOVA and cMy-mOVA-OT-II mice are displayed. A log-rank test (Mantel-Cox) did not indicate a significant difference between the strains. All sham-operated mice of both strains survived until the end of the experiment and are therefore not displayed in the figure.

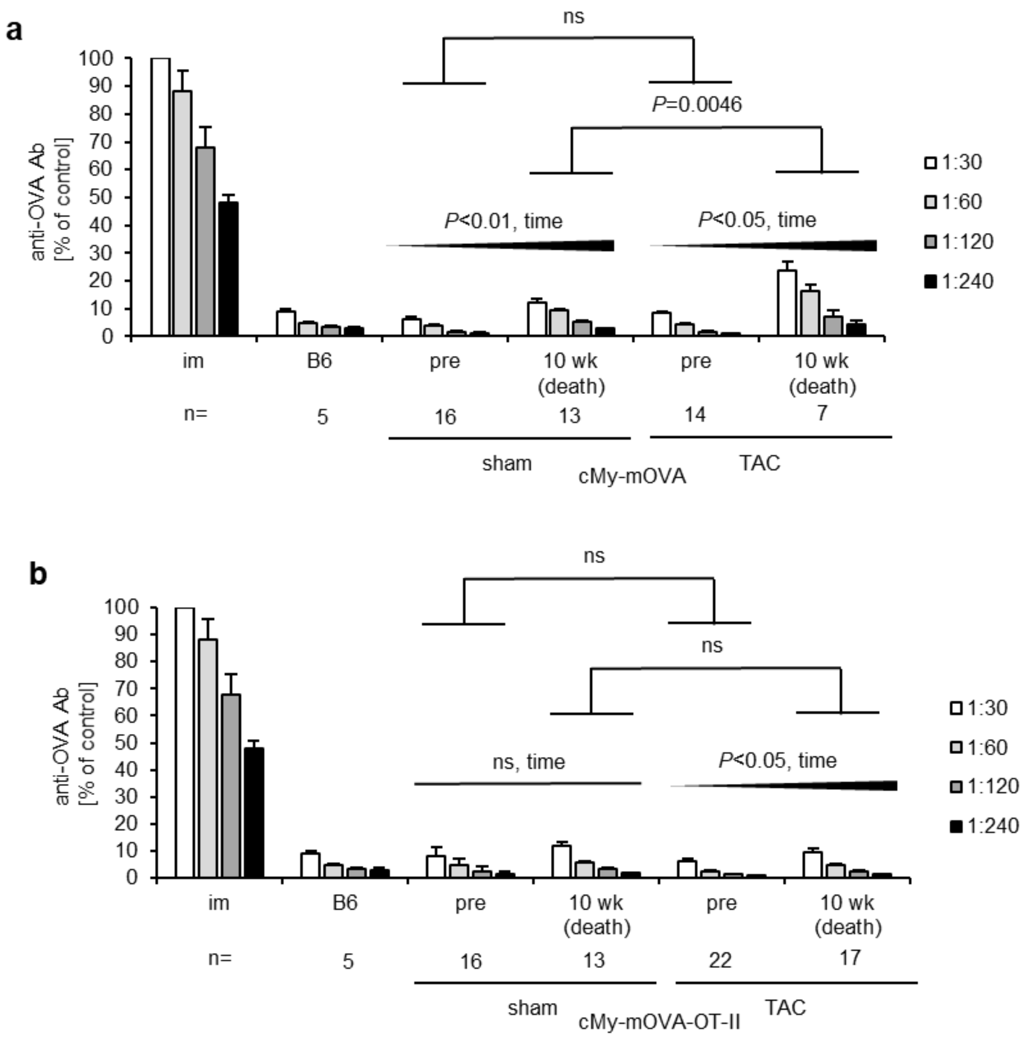

Supplementary Figure 4. Slight induction of OVA-specific autoantibodies in cMy-mOVA mice after TAC. OVA-specific autoantibodies were determined by ELISA in serial-diluted (1:30, 1:60, 1:120, 1:240) sera of (**a**) cMy-mOVA and (**b**) cMy-mOVA-OT-II mice before (pre) and at 10 weeks after sham or TAC surgery when the mice were sacrificed. Pooled serum (pre-diluted 1:10000) of 4 FVB mice immunized with OVA (im) served as positive control on each plate and the OD at the highest dilution was set to 100 % to adjust the other results and means plus SEM are shown. Sera of C57BL/6 (B6) mice (n=5) served as negative control. The numbers of analyzed animals are indicated. The increase of anti-OVA antibodies over time (pre compared to 10 weeks) and differences between sham and TAC-operated mice have been analyzed by repeated measures ANOVA and significant *P*-values are indicated (ns, non-significant).

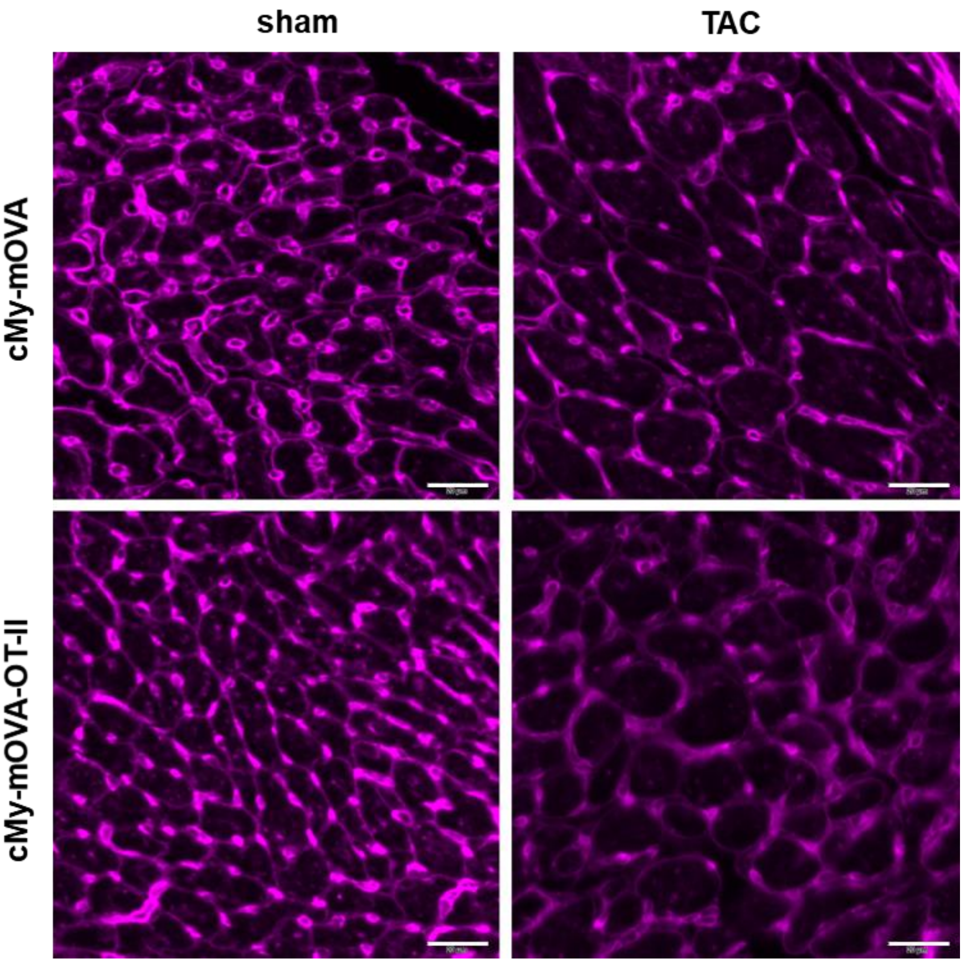

Supplementary Figure 5. Heart sections were stained as exemplified here with Alexa680-labeled WGA, which stains plasma membranes, to determine the CSA of cardiomyocytes in ventricles of sham and TAC-operated cMy-mOVA and cMy-mOVA-OT-II mice. The bars indicate 20 µm.

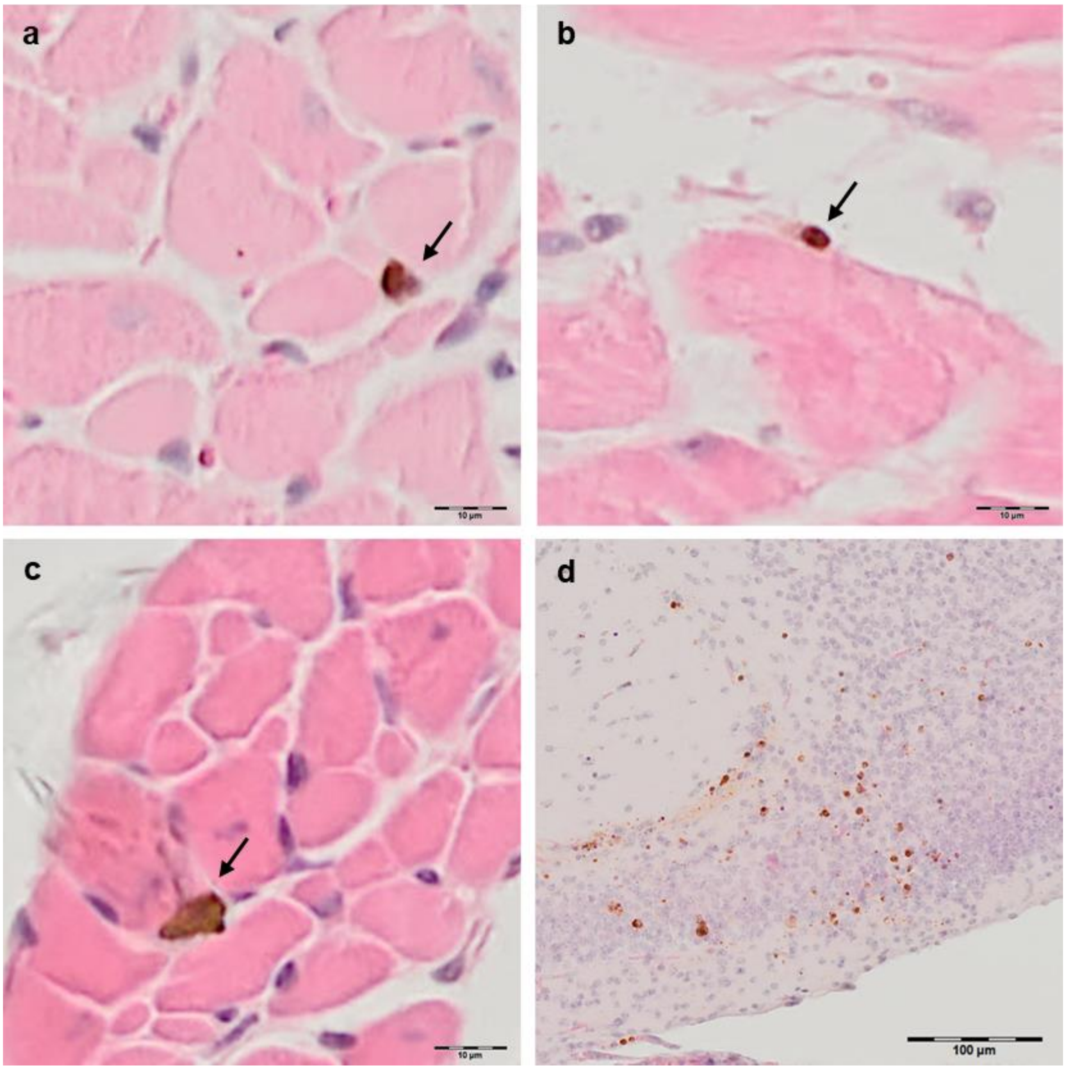

Supplementary Figure 6. In heart sections of TAC-operated mice (**a**-**c**), apoptotic cells expressing cleaved caspase 3 were detected by immunohistochemistry and are marked by an arrow. Whereas most apoptotic cells were non-cardiomyocytes (**a**, **b**), an apoptotic cardiomyocyte (**c**) was detected in a cMy-mOVA mouse. A section of a hypocampus of a 7 days old mouse, which contains several apoptotic cells, is shown as positive control (**d**). The bars indicate 10 µm in panels (**a**-**c**) and 100 µm in panel **d**.

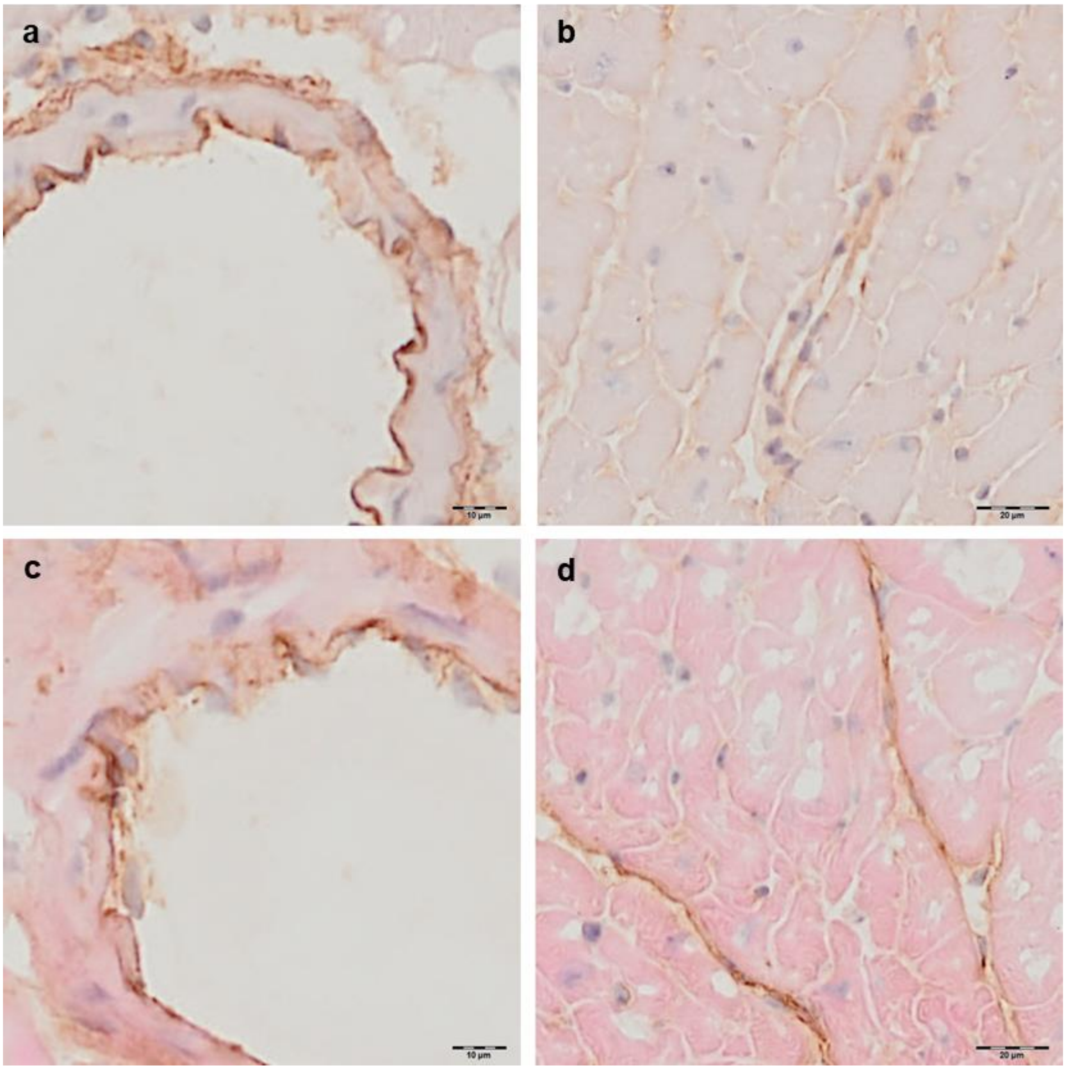

Supplementary Figure 7. In heart sections of cMy-mOVA (**a**, **b**) and cMy-mOVA-OT-II mice (**c**, **d**), endothelial cells expressing CD31 (PECAM-1) were detected by immunohistochemistry. In panels **a** and **c**, the endothelial cells of larger vessels are stained whereas panels **b** and **d** display capillaries in the myocardium. The bars indicate 10 µm in panels **a** and **c** and 20 µm in panel **b** and **d**.

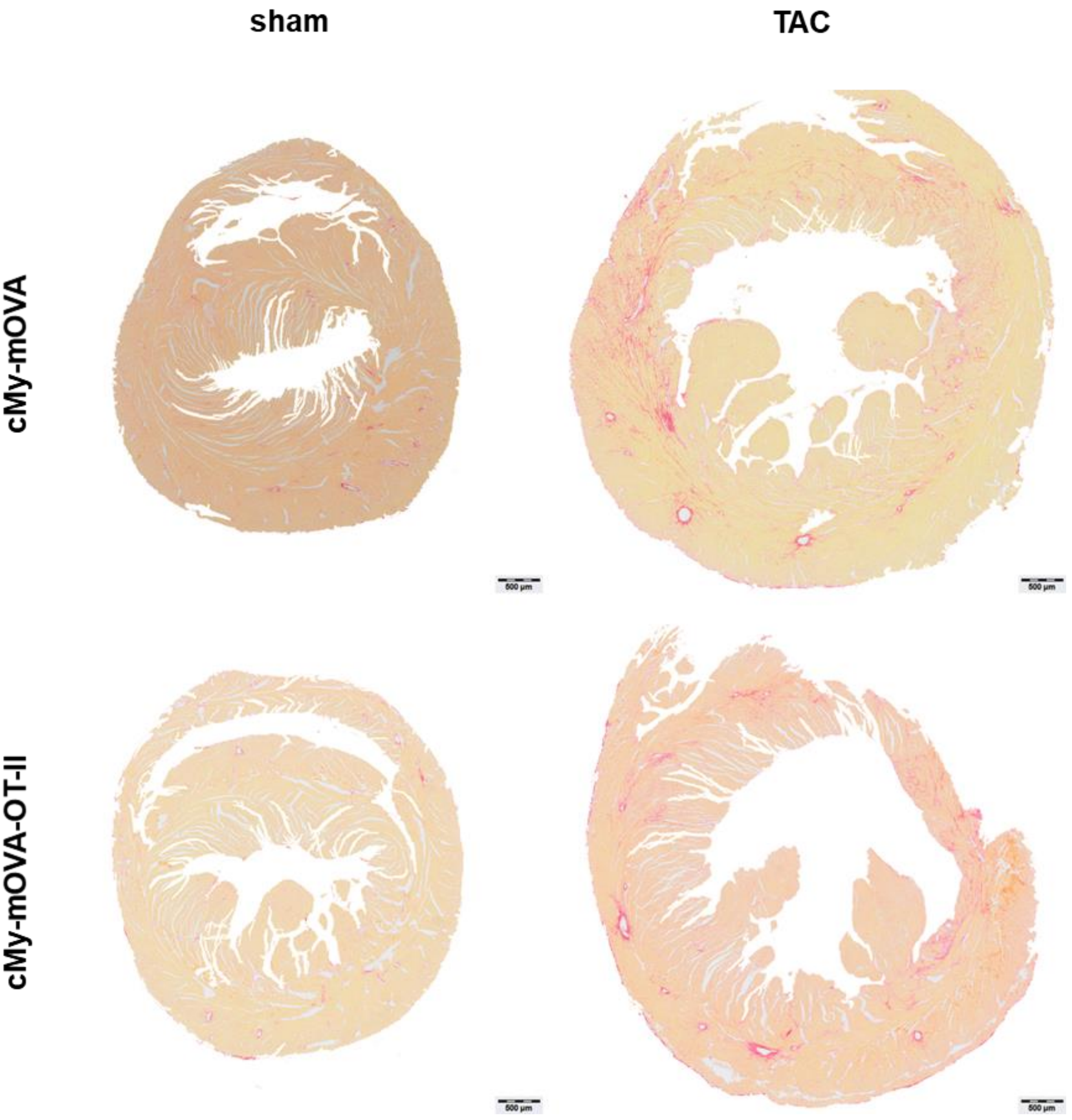

Supplementary Figure 8. Heart sections were stained as exemplified here with Sirius Red, which stains collagen, to determine the fibrotic areas in the ventricles of sham and TAC-operated cMy-mOVA and cMy-mOVA-OT-II mice. Fibrotic areas directly associated with vessels with a diameter of more than 100 µm were considered as perivascular fibrosis. The bars indicate 500 µm.

Supplementary Tab. 1 Antibodies and isotype controls used for flow cytometry

Antigen	Isotype	Clone	Label	Supplier
CD3	rat IgG_{2b}	17A2	FITC<br>PE	Biolegend, Fell, Germany
CD4	rat IgG_{2a}	RM4-5	PE<br>PE/Cy5	Biolegend, Fell, Germany
CD8	rat IgG_{2a}	53-6.7	PE/Cy5	Biolegend, Fell, Germany
CD19	rat IgG_{2a}	6D5	FITC	Biolegend, Fell, Germany
CD25	rat IgG_1	PC61	FITC	Biolegend, Fell, Germany
CD45R/B220	rat IgG_{2a}	RA3-6B2	PE	Biolegend, Fell, Germany
CD49b	rat IgM	DX5	PE	BioLegend, Fell, Germany
CD69	hamster IgG	H1.2F3	PE	BioLegend, Fell, Germany
FOXP3	mouse IgG_1	150D	PE	BioLegend, Fell, Germany
IL-2	rat IgG_{2b}	JES6-5H4	PE	Biolegend, Fell, Germany
IL-4	rat IgG_1	11B11	PE	Biolegend, Fell, Germany
IL-6	rat IgG_1	MP5-20F3	PE	Biolegend, Fell, Germany
IL-17A	rat IgG_1	TC11-18H10.1	PE	Biolegend, Fell, Germany
IL-17F	mouse IgG_1	9D3.1C8	PE	BioLegend, Fell, Germany
IL-10	rat IgG_{2b}	JES5-16E3	PE	Biolegend, Fell, Germany
IFN-γ	rat IgG_1	XMG1.2	PE	Biolegend, Fell, Germany
TCRγδ	hamster IgG	GL3	PE	BioLegend, Fell, Germany
TCRVβ5.1/5.2	mouse IgG_1	MR9-4	FITC	BioLegend, Fell, Germany
TNF-α	rat IgG_1	MP6-XT22	PE	Biolegend, Fell, Germany
TGF-β	mouse IgG_1	TW7-20B9	PE	BioLegend, Fell, Germany
-	hamster IgG	HTK888	PE	BioLegend, Fell, Germany
-	mouse IgG_1	MOPC-21	FITC<br>PE	BioLegend, Fell, Germany
-	rat IgG_1	RTK2071	FITC<br>PE	BioLegend, Fell, Germany

Antigen	Isotype	Clone	Label	Supplier
-	rat IgG_{2a}	RTK2758	FITC<br>PE	BioLegend, Fell, Germany
-	rat IgG_{2b}	RTK4530	FITC<br>PE	BioLegend, Fell, Germany
-	rat IgM	RTK2118	PE	BioLegend, Fell, Germany

The following abbreviations are used: FITC, fluorescein isothiocyanate, PE, phycoerythrin; PE/Cy5, phycoerythrin/cyanine 5.
